# Author Correction: Trans-crustal structural control of CO_2_-rich extensional magmatic systems revealed at Mount Erebus Antarctica

**DOI:** 10.1038/s41467-022-31694-6

**Published:** 2022-07-13

**Authors:** G. J. Hill, P. E. Wannamaker, V. Maris, J. A. Stodt, M. Kordy, M. J. Unsworth, P. A. Bedrosian, E. L. Wallin, D. F. Uhlmann, Y. Ogawa, P. Kyle

**Affiliations:** 1 University of Canterbury, Gateway Antarctica, Christchurch, New Zealand; 2 Institute of Geophysics, Czech Academy of Science, Prague, Czech Republic; 3 University of Utah, Energy & Geoscience Institute, Salt Lake City, UT USA; 4 Numeric Resources LLC, Salt Lake City, UT USA; 5 University of Alberta, Department of Physics, Edmonton, AB Canada; 6 United States Geological Survey, Denver, CO USA; 7 University of Hawaii at Manoa, Hawaii Institute of Geophysics and Planetology, Honolulu, HI USA; 8 First Light Mountain Guides, Chamonix, France; 9 University of Lausanne, Department of Earth Science, Lausanne, Switzerland; 10 Tokyo Institute of Technology, Volcanic Fluid Research Centre, Tokyo, Japan; 11 New Mexico Institute of Mining and Technology, Socorro, NM USA

**Keywords:** Volcanology, Geophysics, Tectonics, Volcanology, Geophysics, Tectonics

Correction to: *Nature Communications* 10.1038/s41467-022-30627-7, published online 30 May 2022.

The original version of this Article contained an error in Figure 5, which read DFGZ instead of DGFZ. This has been corrected in both the PDF and HTML versions of the Article.

