# Supplementary material for: Trans-crustal structural control of CO2-rich extensional magmatic systems revealed at Mount Erebus Antarctica
Source: Nat Commun. 2022 May 30;13:2989. doi: 10.1038/s41467-022-30627-7 (PMC9151792; doi:10.1038/s41467-022-30627-7)
Supplement: Supplementary file 1 — Supplementary Material [file 41467_2022_30627_MOESM1_ESM.pdf]

Supplementary Information:
Trans-crustal structural control of CO_2-rich extensional magmatic systems revealed at Mount Erebus Antarctica

G.J. Hill[1,2], P.E. Wannamaker[3], V. Maris[3], J.A. Stodt[4], M. Kordy[3], M.J. Unsworth[5], P.A. Bedrosian[6], E.L. Wallin[7], D.F. Uhlmann[8,9], Y. Ogawa[10], & P. Kyle[11]

[1]*University of Canterbury, Gateway Antarctica, Christchurch, New Zealand*
[2]*Institute of Geophysics, Czech Academy of Science, Prague, Czech Republic*
[3]*University of Utah, Energy & Geoscience Institute, Salt Lake City, UT, USA*
[4]*Numeric Resources LLC, Salt Lake City, UT, USA*
[5]*University of Alberta, Department of Physics, Edmonton, AB, Canada*
[6]*United States Geological Survey, Denver, CO, USA*
[7]*University of Hawaii at Manoa, Hawaii Institute of Geophysics and Planetology, Honolulu HI, USA*
[8]*First Light Mountain Guides, Chamonix, France*
[9]*University of Lausanne, Department of Earth Science, Lausanne, Switzerland*
[10]*Tokyo Institute of Technology, Volcanic Fluid Research Centre, Tokyo, Japan*
[11]*New Mexico Institute of Mining and Technology, Socorro, NM, USA*

Contact: *gjhill@ig.cas.cz*

Supplementary Methods
Magnetotelluric Method
Temporal fluctuations in the naturally occurring, external magnetic field induce electrical currents in the Earth. The MT method exploits these induced electrical currents to determine the resistivity structure of the crust and upper mantle[1]. From simultaneous measurements of the surface components of the electric (**E**) and horizontal magnetic (**H**) fields, a 2x2 transfer function, the impedance tensor **Z**, is determined in the frequency domain and defined by relationship $\mathbf{E} = \mathbf{Z}\cdot\mathbf{H}$. Similarly, the relationship between the vertical magnetic field H_z and the horizontal magnetic field is expressed in the form of a 1x2 induction transfer function **K** so that $H_z = -\mathbf{K}\cdot\mathbf{H}$. The impedance and induction transfers are complex functions of Earth's resistivity ρ (or conductivity $\sigma = 1/\rho$). The distribution of the resistivity is estimated using inversion modelling[2-4] of one or both of **Z** and **K**. Data collection, response function estimation, and inversion image construction are described further below.

Field Measurement Procedure
MT soundings were collected with commercially available Phoenix V5-2000 MT systems, using a '+' array layout for the electric dipoles. Expanded CP grade 2 titanium tubular slit sheets (45 x 60 cm) chosen for their electrochemical stability were used for the electric field measurements[5] (Figure S1). High-moment induction coils were used for the magnetic fields. Sites were accessed primarily by helicopter (Eurocopter AS350B2 & AS350B3, and Bell 212); a small number of sites were accessed using overland travel via Hagglund or snowmobile. Recording times typically varied from 3 to 6 days, though logistical and weather constraints resulted in recording times of up to 12 days. Sensor axes were aligned with geomagnetic coordinates to minimize orientation errors during setup; subsequently all soundings were rotated to x = Mercator north (Meridian 168°E) coordinate system as in Figure 1 for subsequent display and analysis. Individual station spectral rotations were computed using the NOAA on-line calculator for the date of station installation and ranged between -139.8° and -142.6°.

Essentially the same procedure of our previous campaigns[2,5-8] was employed to achieve high-fidelity electric field measurements in the face of the large contact resistances R_c represented by

the electrode-firn interface, which for Mount Erebus lay in the 0.5 to 2 MΩ range. Any shunt capacitance C_{sh} acting after R_c and before a receiver input will generate filter effects that can affect the E-field measurements at MT frequencies. A model of C_{sh} caused by distributed wire-to-ground capacitance[2,6,8,9] demonstrates how such filter effects can be mitigated by inserting in series as close to the electrode as possible an amplifier with high input impedance subsequently connected to the receiver signal input (Figure S1).

A properly designed amplifier will buffer R_c and deliver its output voltage, ideally a copy of the desired signal but now with low series output resistance (typically <100Ω) to the receiver input. Shielded twisted pair bipole cable connects the E-field buffer amplifier signal and ground outputs to the receiver signal and ground inputs (Figure S1). The cable shielding (not shown on Figure S1) is connected to the receiver ground only, and left unconnected at the buffer output. Although the filter effects due to E-bipole C_{sh} are greatly mitigated by the low series output resistance of the buffer acting in lieu of R_c there is still some shunt capacitance acting at the input of the buffer amplifier (typically, C_{sh} = 20-30pf) which could affect data at frequencies higher than ~100 Hz at the R_c experienced on Mount Erebus. This may be due to inherent op-amp input capacitance and to other parasitic capacitance including that of a short pigtail wire connecting the amplifier input to the electrode, that can react with R_c.

Non-Planar and Other Signal Outlier Removal

The MT method makes the fundamental assumption that the incident EM source field is a vertically propagating horizontally polarized plane wave. The validity of the plane wave assumption may be challenged in polar regions due to the concentration of the magnetospheric currents generated by the solar wind during periods of elevated activity, causing field aligned currents to pass into and out of the polar auroral oval[10-12]. We avoid non-planar effects with a three-step approach, similar to our recent work in the Transantarctic Mountains[2]. First, Ap magnetic activity indices[13] were compiled for all three field campaigns (Figure S2), showing predominantly low activity levels of Quiet and Unsettled. The low levels suggest few anomalous current systems within the auroral oval throughout the data collection[6].

Secondly, spectral strength was analysed as a function of time (Figure S3). A diurnal cyclicity in ionospheric signal strength is present, with higher intensity intervals centered around local midnight. The higher intensity periods result from a reduced distance to the more intense night-time auroral oval segment, the cyclicity constituting the polar electrojet solar quiet variation[2,6,10]. The steady nature of cyclic variation in ionospheric signal strength indicates a lack of major solar magnetic disturbances that generate magnetospheric substorms leading to non-planar effects.

Third, the cyclic signal behaviour itself was tested to determine if it could represent non-planar contributions by subdividing the total recording into 12-hour segments centered on the highest and lowest activity time windows[2]. The high and low energy data were grouped and independent MT soundings calculated (Figure S4), using robust remote reference processing[14], representing the two signal activity levels. The low and high activity level soundings (Figure S4), apart from minor scatter, have the same primary character over the entire period band; similar results were determined for other soundings that straddled the increased magnetic activity periods. The independence of the MT soundings to variations in the ionospheric signal strength indicates that source effects are negligible, and the entire time series hence was used to determine the MT responses used in the analysis and modelling. A dedicated reference site recording both **E** and **H** components was run (for all three seasons) on Hut Point peninsula ~25 km from Scott

Base/McMurdo Station. The relatively high strength of the electric field at the shortest periods lends it an advantage in remote referencing that portion of the response[2,6].

Impedance Phase Tensor and Invariant Apparent Resistivity Plots

Impedance phase ellipses derived from the observed impedance were introduced with Figure 1 in the main text at a period of 10.7 s. Phase ellipse plan views at four diagnostic periods approximately one decade apart are plotted in Figure S5. At the shortest period of 0.067 s, high values of Φ_2 exceeding 60° (warm colours) are characteristic, indicating passage from a resistive near-surface stratum into a conductive layer just below. At 0.89 s, still reflecting upper crustal structure, the higher values begin to concentrate from Mount Erebus westward suggesting that the causative low resistivity medium is thicker there. At 7.1 s, the ellipses of highest phase values clearly group in the southwest sector of the island from Mount Erebus. Understanding the depth extent of the presumed low resistivity structure there requires quantification by formal 3D inversion. The ellipse colours at 57 s are subtler and cooler, but now higher values (light blue-green) with large ellipticity lie along the slope of Mount Erebus almost directly west of the summit crater. This is taken to indicate deep structure associated with the magma system.

Complementary to the phase ellipse data plots are multi-period apparent resistivity "spot" plots using the invariant impedance $Z_{ivt} = (Z_{xy}-Z_{yx})/2$ (Figure S6). At the shortest period T = 0.0083 s, ρ_{ivt} commonly reaches 1000 Ωm except for the sites off the southern coast of Ross Island over seawater. The high values are compatible with shallow, unaltered volcanic flows. At the next longer period of 0.067 s, values over the eastern half of Ross Island and along its western coast are somewhat lower indicating that the MT signals are detecting lower resistivity below those flows. By 0.89 s, ρ_{ivt} is lower more uniformly over the island except perhaps for much of the summit plateau. This trend continues for T = 7.1 and 57 s, with the eastern half of the island not quite as conductive apparently. However, by 455 s, the lowest ρ_{ivt} values around 10 Ωm are concentrated in the southwest sector of Ross Island where the greatest accumulation of altered volcanic flows resides. Response of deeper magmatic structure will have these first order variations superimposed and so show up more clearly in the phase ellipses. However, we do point out the highly variable values at the longer periods over short distances around Erebus crater, with generally very high ρ_{ivt} on the east side and low ρ_{ivt} on the west side of the crater.

Observed induction arrows are plotted in Figure S7 for the convention where real components of the arrows point toward conductors. At the shortest periods, real arrows of moderate magnitude point toward the summit plateau, while on the plateau itself the magnitudes are small. As periods increase beyond several seconds, real arrows of substantial magnitude form pointing toward the seawater surrounding Ross Island plus an indeterminate amount of marine sediments. At the very longest periods, several hundreds of seconds, outward arrow orientation is still apparent although orientations and magnitudes have become less coherent reflecting noise in the long period vertical magnetic field transfer function.

Inversion Imaging Procedure

Three-dimensional (3D) simulation and inversion of the Erebus MT responses uses the HexMT algorithm[2-4], which implements direct solvers and deformable hexahedral edge finite elements (FE) to precisely incorporate topography (Figure S8). MT responses from 129 stations were input to HexMT using data over the wave period range of 0.0083 to 910 s which represented the highest quality results. Because we could not define good constraints on possibly thick, low-resistivity sedimentary sections offshore, and because the MT vertical magnetic field transfer

function is comprised entirely of secondary vertical magnetic field variations sensitive to lateral changes, the induction arrow data are downweighted. Errors floors on the real and imaginary parts of the complex impedance elements Z_{ij} were applied with ***max*** (assigned Z_{ij} error, 5%($|Z_{xy}-Z_{yx}|/2$)) at each frequency. Ross Island elevations and surrounding bathymetry were supplied from a resource compiled by the New Zealand GNS Science and the outmost FE surface elevations were fixed to 0 m. The FE mesh consisted of 127(x) by 193(y) by 66(z) cells with 14 layers of air. The smallest cell widths in the center of the MT data coverage were 100 m while the thinnest cells at the surface were 40 m, growing by 15% per element with depth. Apart from a two-element rim around the mesh edge and the fixed air and seawater resistivities, all elements were inversion parameters for a total of 1031577. Seawater was assigned a fixed 0.3 Ωm resistivity[15], air at 10^{18} Ωm, while the earth starting resistivity was 100 Ωm from the surface to the base of the mesh at 660 km depth. Ice cells started at 600 Ωm but were allowed to vary as inversion parameters.

A phase tensor representation of the initial, 100 Ωm finite element mesh MT response appears in Figure S9 for the same four periods of observed responses as in Figure S5. Colour variations are subtle compared to the range in the observed ellipses and represent mainly the effect of Ross Island's topography on the MT response. At short periods, yellowish coloured ellipses cluster near Erebus summit and to a lesser extent Mount Terror. At the longer periods, the warmer colours are more widespread, for instance all along the E-W oriented spine of Ross Island. These warmer colours correspond with impedance magnitudes falling as period increases as the peaked topography causes a reduction of electric field over elevation increases[3]. For the few sites off the south shore over seawater, short period ellipses with warm colours indicate MT sensing the seawater below ice, while cool colours at longer periods make evident the lithology below the seawater.

Apparent resistivities constructed from the impedance invariant Z_{xy}-Z_{yx} (Figure S10) show decreases toward longer periods from the host value of 100 Ωm to as low as 20 Ωm over Mount Erebus area particularly. This is expected to cause commensurate distortions in the inversion model structure if not explicitly accounted for as in our finite element mesh[3,4]. Only around the very margins of the island at long periods is there a noticeable increase in ρ_{ivt} due to the resistive island surrounded by conductive seawater, accompanied by phase ellipse values at middle periods being slightly below 45°. We have run a flat-earth version of the Ross Island finite element starting model to isolate the coast effect (not plotted) and it confirms this inflation in ρ_{ivt} is confined to such a narrow strip ~5 km in from the coast.

Computed induction arrows for the starting model appear in Figure S11. Here it is apparent that the short period induction arrows pointing toward the summit plateau can be explained mostly by the topography itself. A flat Ross Island calculation (not plotted) shows essentially zero induction vector length in this vicinity. At longer periods, the seacoast effect grows. It is smoother than the observed data, which is unsurprising with the expectation that crustal heterogeneity is present. West of the summit plateau at middle periods of 7.1 and 56.9 s, the observed induction arrows appear significantly smaller than those of the starting model, suggesting some conductive structure is competing with the seacoast effect in that area.

Because of the inability to constrain the effect of large possible volumes of conductive marine sediments surrounding Ross Island, and the fact that the induction arrow vertical magnetic field is secondary, we have emphasized inversion models computed using the four complex elements of the impedance only. This is distinct from highly constrained vertical magnetic field transfer

function inversions where the seawater is assumed to be the only lateral heterogeneity and the interpreted resistivity below an island is of idealized (e.g. 1D) form[16]. Inversion convergence for the impedance was essentially monotonic from a starting nRMS misfit value of 22.4 to a final value of 1.29 in 18 iterations (Figure S12) for the preferred resistivity model (Figures 2-4). A measure of model response fit to the data at individual MT sites appears in Figure S13 using a "spot" plot with colour coding of nRMS at each site. Fit over the sites is generally uniform apart from a few around the summit crater which show scatter apparently greater than the nominal error bars. Computed phase ellipses and invariant apparent resistivity ρ_{ivt} are shown in Figures S14 and S15 and simulate the observed response behaviour in good detail. Figures S6 and S15 illustrate just how wide-ranging the resistivity structure is over very short distances across the crater.

Example sounding curves west and east of Erebus summit crater are shown together with inversion model computed responses (Figure S16). The western site shows relatively high impedance phases and falling apparent resistivities toward the longer periods relative to the eastern, signifying the low deep crustal resistivity west of the summit. Computed induction arrows (Figure S17) appear largely consistent with the data despite not being explicitly included in the inversion. Minor differences include some irregularity in arrow direction surrounding the summit at short periods. In addition, the observed magnitudes are somewhat smaller than computed elsewhere at short periods and somewhat larger than computed near the shoreline at medium to longer periods.

Joint Impedance-Vertical Magnetic Field Transfer Function Inversion

Vertical magnetic field transfer function data are included conservatively in a trial inversion by limiting it to periods shorter than 140 s with an error floor of 0.075. Computed induction arrows at short to middle periods are shown in Figure S18 for comparison to the observed data (Figure S11) and the impedance inversion response (Figure S17). Second order but noticeable improvements in fit to the observations can be seen, such as more regular behaviour of the arrows around the summit area and a few of the magnitudes closer to the shoreline. Plan views of the model compared to that from impedance (Figure S19) show only minor differences. The structure around the summit area is somewhat smoother and some of the streakiness south of the summit area is slightly reduced. The mid-crustal structural features are essentially identical, as is the case for deeper structures (not shown). At deep-crustal levels and beyond it is difficult to know what structural differences would be resulting from poor offshore constraints and so we do not dwell on those levels regarding vertical magnetic field transfer function influence.

Inversion Model Testing

The ill-posed nature of inversion modelling for diffusion-based electromagnetics such as MT warrants testing. First, we clarify the causative structure for the zone of phase ellipses that suggest a mid-crustal conductor below the island sector southwest of Mount Erebus summit (Figures 1 & S5c). To do this, we take the preferred model (Figures 2-4) and progressively truncate it at depth and show the resultant phase ellipse responses (Figure S20). The first such is at the Moho, near 23 km depth[17,18]. The second is at a depth of 7 km in the upper middle crust. One sees that the computed ellipses in Figure S16 change almost imperceptibly with such truncations, strongly indicating that the phase ellipse Φ_2 values at 10.7 s exceeding 45° in the southwest quadrant of the island represent a stack of several kilometers of altered, conductive volcanic flows.

We also examine results of this test on longer period phase ellipses (Figure S21). In this case, truncating the inversion does have a noticeable effect. Even with the 23 km constraint, ellipses to the west of Erebus crater become coherently less warm, less extremely elliptical, and more uniformly oriented. When structure is truncated at 7 km, the ellipses lose most of their lateral character indicating that deep crustal structure at the very least is likely present.

Petrological modeling[19,20] of the Erebus system implies phonolitic magma reservoir compositions in the 4-7.5 km depth range and temperatures near 1000°C, with less evolved phono-tephite magma at somewhat higher temperatures at greater depths and a modeled water content of no more than 0.5 wt %. Phonolitic melts with 1.1 wt % H_2O at 1050°C have a measured resistivity[21] of ~0.3 Ωm. Thus, the substantial low resistivity zone in the middle crust has properties approximating pure melt, and this implication is more pronounced for the case of the constrained inversion model. Given its more extreme characteristics, a purely crustal anomalous structure is considered less likely from physical property considerations as well as from explicit inversion testing (Figures 4 and S22), and so an extension into the upper mantle is preferable. We advocate further measurements on the sea ice especially over the relatively shallow Erebus Bay region (southwest of study area) in an effort to improve resolution of the deeper portions of the magmatic system.

For completeness, four additional 3D views are provided (Figure S23). The turn in the low resistivity body in the deep middle crust receives additional perspective in the plot, even though the precise degree of extension of the low resistivity into the upper mantle remains somewhat conjectural. Furthermore, in Figures S24-S26, we show Figures 2-4 of the main text in an alternate colour scheme from the main spectral colour representation.

Supplementary References:

1 Chave, A. D. & Jones, A. G. The Magnetotelluric Method: Theory and Practice. *Cambridge University Press*, doi:10.1017/CBO9781139020138 (2012).

2 Wannamaker, P., Hill, G., Stodt, J., Maris, V., Ogawa, Y., Selway, K., Boren, G., Bertrand, E., Uhlmann, D., Ayling, B., Green, A.M., & Feucht, D. Uplift of the central Transantarctic Mountains. *Nature Commun.* **8**, 1–11 (2017).

3 Kordy, M., Wannamaker, P., Maris, V., Cherkaev, E. & Hill, G. 3-D magnetotelluric inversion including topography using deformed hexahedral edge finite elements and direct solvers parallelized on SMP computers - Part I: Forward problem and parameter Jacobians. *Geophys. J. Int.* **204**, 74–93 (2016).

4 Kordy, M., Wannamaker, P., Maris, V., Cherkaev, E. & Hill, G. 3-dimensional magnetotelluric inversion including topography using deformed hexahedral edge finite elements and direct solvers parallelized on symmetric multiprocessor computers - Part II: Direct data-space inverse solution. *Geophys. J. Int.* **204**, 94–110 (2016).

5 Wannamaker, P.E., Stodt, J.A., & Olsen, S.L. Dormant state of rifting below the Byrd Subglacial Basin, West Antarctica, implied by magnetotelluric (MT) profiling. *Geophys. Res. Lett.* **23**, 2983–2986, doi:10.1029/96GL02887 (1996).

6 Hill, G.J. On the Use of Electromagnetics for Earth Imaging of the Polar Regions. *Surv. Geophys.* **41,** 5-45 (2020).

7 Wannamaker, P.E., Stodt, J.A., Olsen, S.L., Pellerin, L., & Hall, D., Structure and thermal regime beneath the South Pole region, East Antarctica, from magnetotelluric measurements. *Geophys. J. Int.* **157**, 36-54 (2004).

8 Wannamaker, P. E., Stodt, J. A., Hill, G. J., Maris V., & Kordy, M. A. Thermal regime and state of hydration of the Antarctic upper mantle from regional-scale electrical properties: in The geochemistry and geophysics of the Antarctic mantle. *Geol. Soc. Lon. Mem.* **56**, eds. A. Martin & W. van der Wal, 14 pp., doi:10.1144/M56-2020-4 (2021).
9 Zonge, K.L., & Hughes, L.J. Effect of electrode contact resistance on electric field measurements. *in: SEG Annual Meeting Abstracts* **MIN 1.5**, 231-234 (1985).
10 Akasofu, S. Physics of magnetospheric substorms. *Astrophys. Space Sci. Lib.* **47**, (D. Reidel, Amsterdam, 1977).
11 Rostoker, G. Phenomenology and physics of magnetospheric substorms. *J. Geophys. Res.* **101**, 12955–12973 (1996).
12 McPherron, R. Early studies in solar wind coupling and substorms. *Journal of Geophysical Research: Space Physics*, 125, e2019JA027615., doi.org/10.1029/2019JA027615 (2020).
13 Zhizhin, M., Kihn, E., Redmon, R., Medvedev, D., & Mishin, D. Space physics interactive data resource - SPIDR. *Earth Sci. Inform.* **1**, 79–91 (2008).
14 Jones, A. G., Chave, A. D., Egbert, G., Auld, D., & Bahr, K. A comparison of techniques for magnetotelluric response function estimation. *J. Geophys. Res.* **94**, 14201-14213 (1989).
15 Filloux, J. H., Instrumentation and experimental methods for oceanic studies: in Geomagnetism, 1, ed. by J. A. Jacobs, 143-248, *Academic Press*, San Diego (1987).
16 Morschhauser, A., Grayver, A., Kuvshinov, A., Samrock, F., and Matzka, J. Tippers at island geomagnetic observatories constrain electrical conductivity of oceanic lithosphere and upper mantle. *Earth, Planets and Space,* **71**, 9 pp., doi:10.1186/s40623-019-0991-0 (2019).
17 Lawrence, J. F., Wiens, D. A., Nyblade, A. A., Anandakrishnan, S., Shore, P. J., & Voigt, D. Crust and upper mantle structure of the Transantarctic Mountains and surrounding regions from receiver functions surface waves, and gravity: Implications for uplift models. *Geochem. Geophys. Geosys.* **7**, Q10011 doi:10.1029/2006GC001282 (2006).
18 Finotello, M., Nyblade, A., Julia, J., Wiens, D., & Anandakrishnan, S. Crustal Vp–Vs ratios and thickness for Ross Island and the Transantarctic Mountain front, Antarctica. *Geophys. J. Int.* **185**, 85–92, doi:10.1111/j.1365-246X.2011.04946.x (2011).
19 Oppenheimer C., Moretti R., Kyle P. R., Eschenbacher A., Lowenstern J. B., Hervig R. L., & Dunbar N. W. Mantle to surface degassing of alkalic magmas at Erebus volcano, Antarctica. *Earth Planet. Sci. Lett.* **306**, 261–271 (2011).
20 Moussallam, Y., Oppenheimer, C., Scaillet, B., & Kyle, P. Experimental phase equilibrium constraints on the phonolite magmatic system of Erebus volcano, Antarctica. *J. Petrology* **54**, 1285-1307, doi:10.1093/petrology/egt012 (2013).
21 Manning, C. E., Fluids of the Lower Crust: Deep Is Different. *Annu. Rev. Earth Planet. Sci.* **46**, 67-97, doi:10.1146/annurev-earth-060614-105224 (2018).

Supplementary Figures:

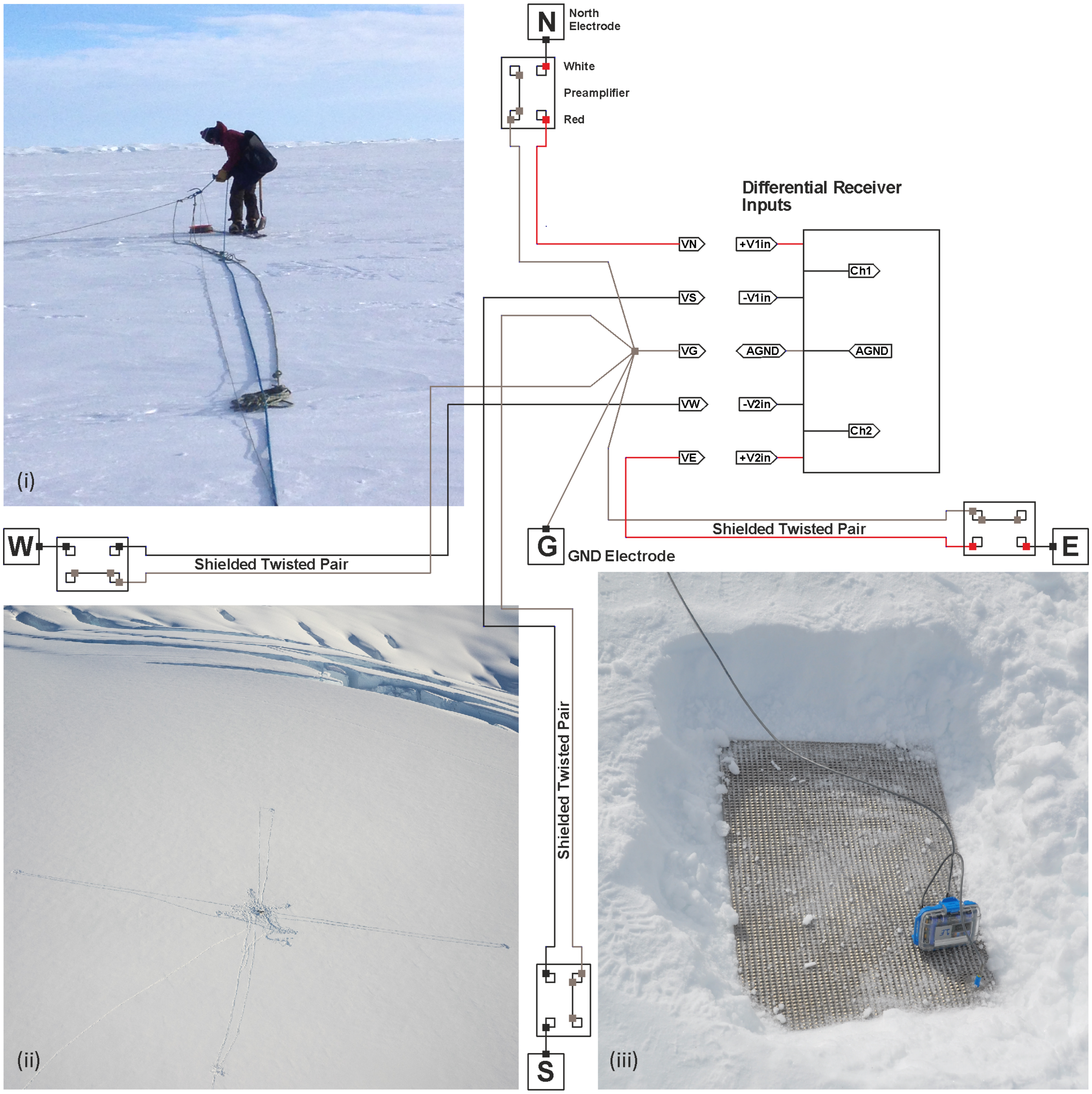

Supplementary Figure S1: Schematic layout diagram of MT site deployment using preamps for high contact resistance environments[6]; (a) photograph of single-person rope travel on skis for electrode installation (photograph P. Bedrosian); (b) aerial photograph of the installed site above a crevasse field on Mount Erebus (photograph D. Uhlmann); (c) photograph of a titanium electrode connected to a preamp prior to burial (photograph K. Selway).

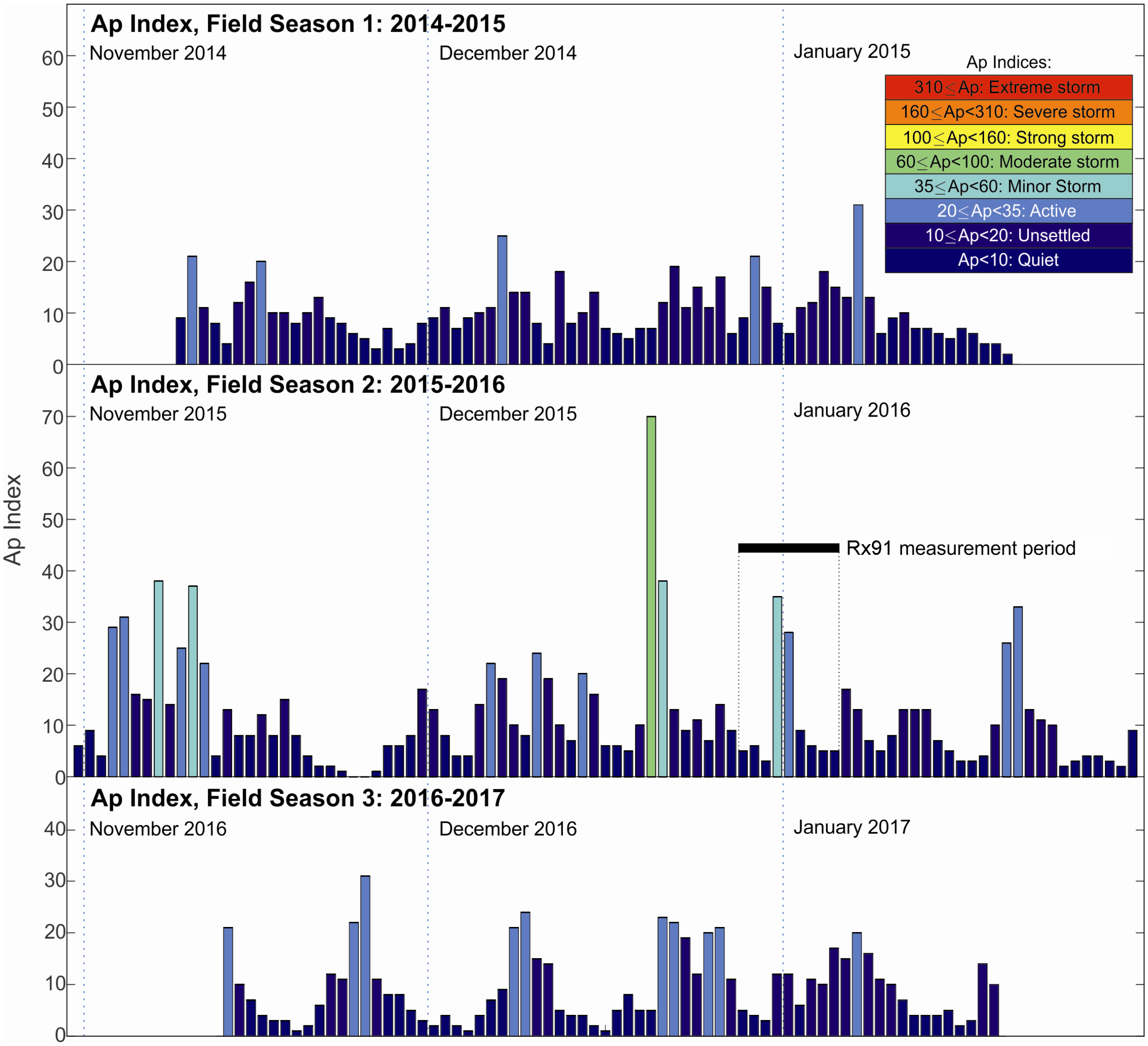

Supplementary Figure S2: Ap indices for all three seasons. Recording window of sounding Rx91 (location marked on Figure S13) used to demonstrate adherence to the plane-wave source requirement of MT (Figures S3 & S4) is marked during the 2015-16 season.

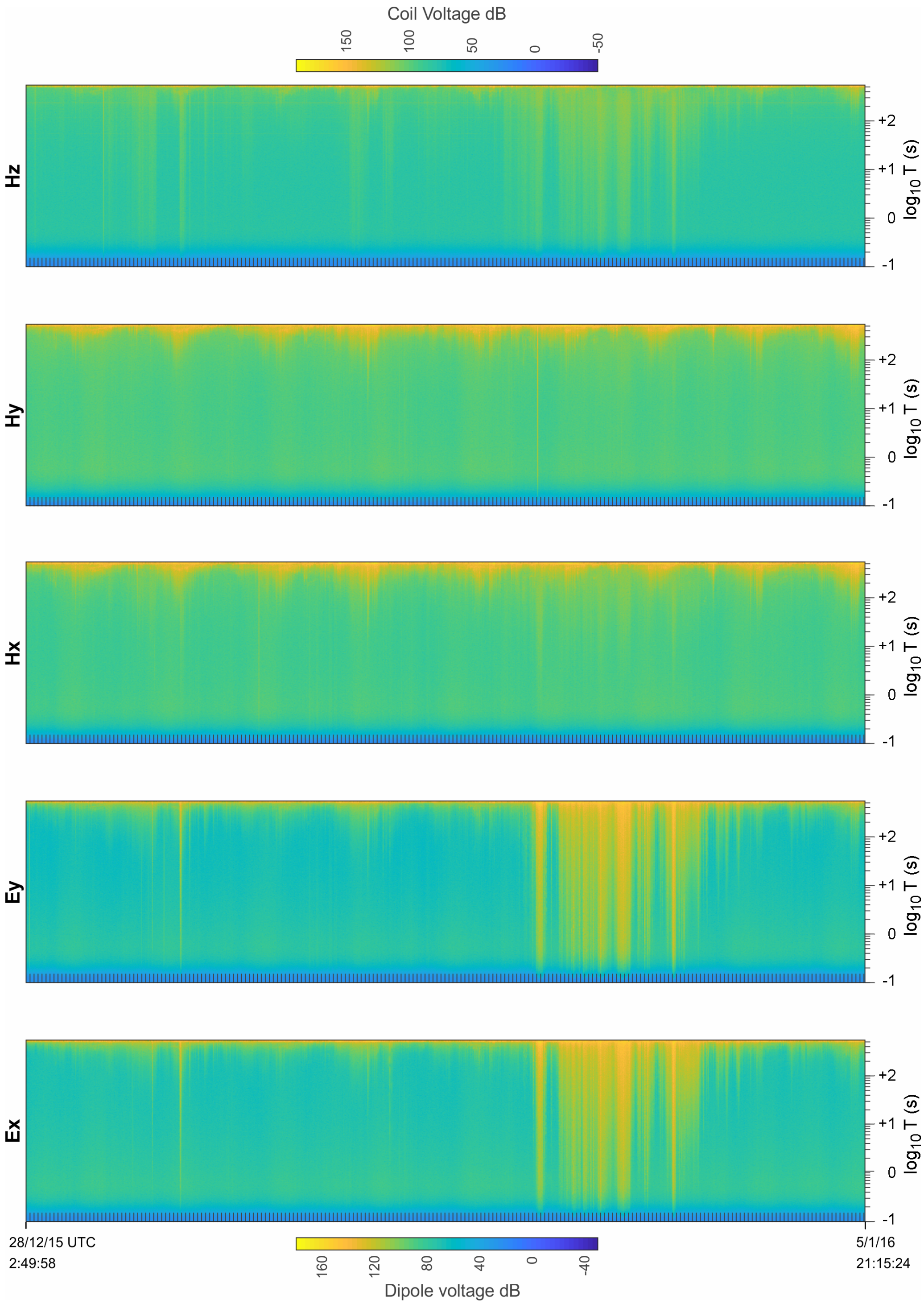

Supplementary Figure S3: Spectra showing cyclic nature of signal strength for sounding Rx91 (location marked on Figure S13). The ~12-hour diurnal high and low energy signal cycle due to Earth rotation beneath the polar electrojet oval is clearly seen in the H_x and H_y channels and to a lesser extent in the record of E_x and E_y. A large storm system with strong winds ca. Jan 2-4 2016 can be seen by the high energy response in both the electric field channels and to a lesser extent on H_z.

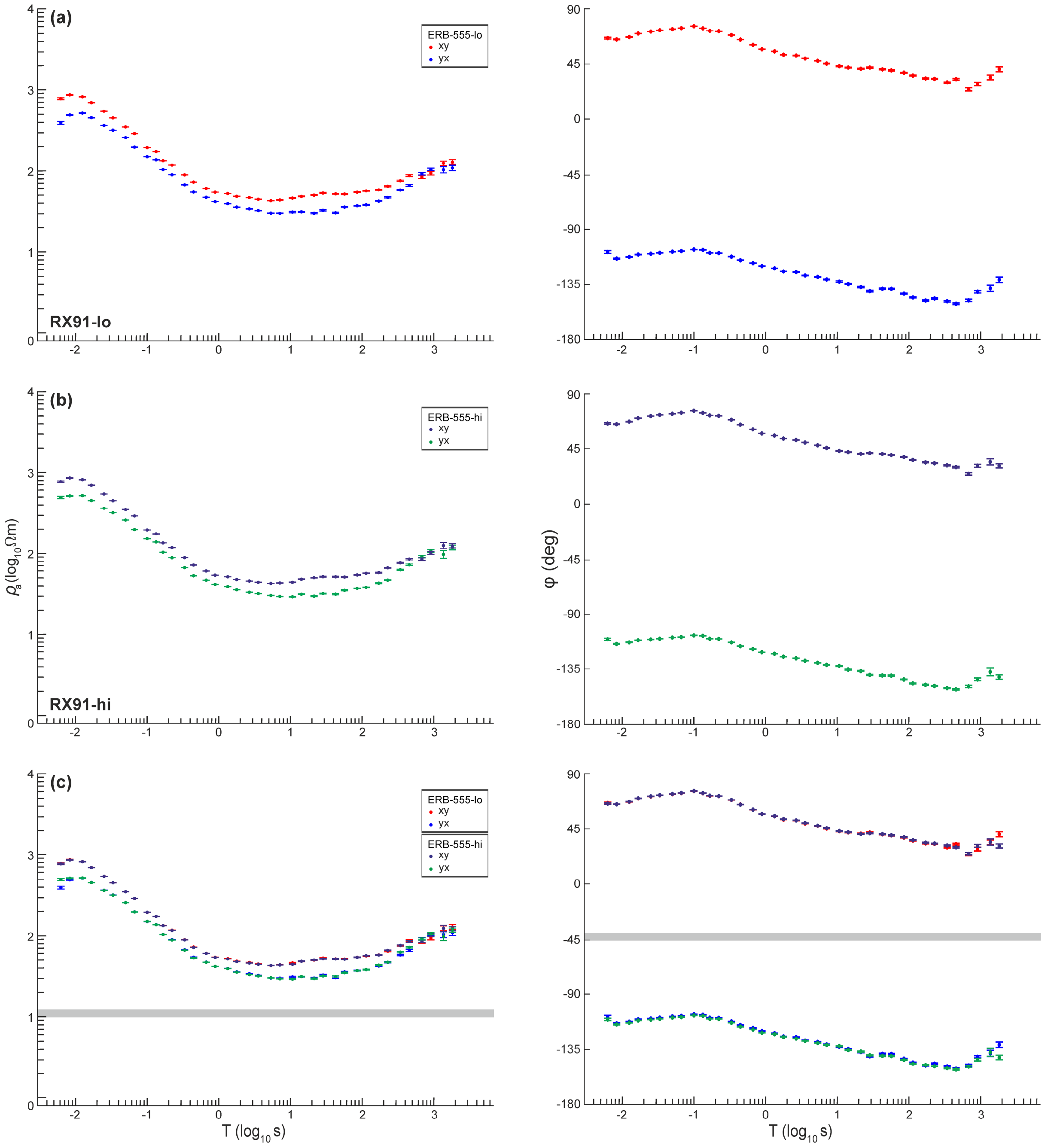

Supplementary Figure S4: MT responses for sounding Rx91 (location marked on Figure S9) on the southern flanks of Mount Terror determined using separated low (a) and high (b) energy signal time windows identified in the spectral response (Figure S3). The computed responses from both the independent high and low energy intervals recover nearly identical results as shown in the overlaid view (c). The horizontal grey bars placed arbitrarily at 10 Ωm and -45° depict the error floors used during the inversion, which exceed the difference between the high and low energy interval responses down at least to 900 s period as used in our inversions. This indicates that significant non-plane wave effects are not present, such that the entire recording time can be used in determination of the MT transfer functions.

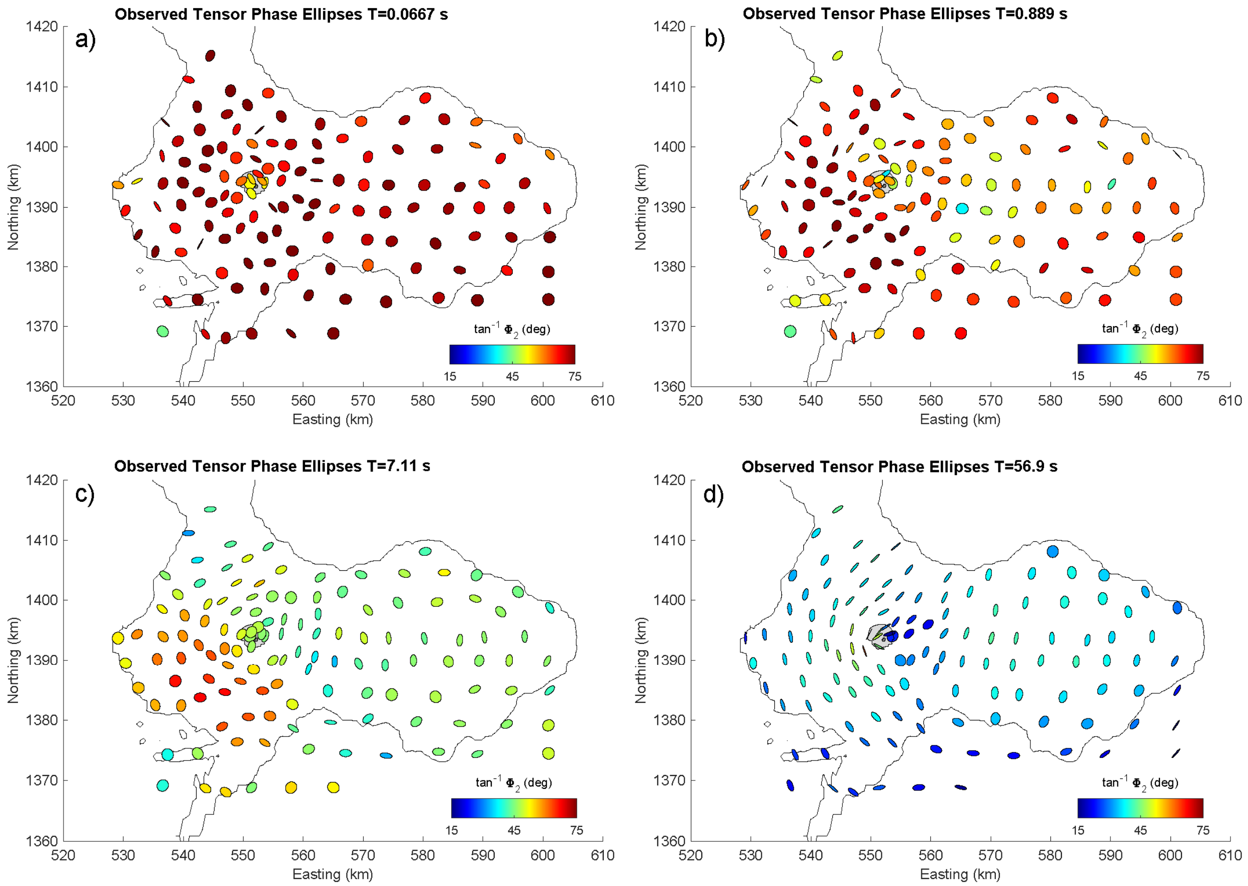

Supplementary Figure S5: Observed phase tensor ellipses at four periods for all 129 stations of the Mount Erebus/Ross Island MT survey. Light grey filled outline denotes Erebus summit plateau while smaller medium grey filled outline denotes the Erebus modern crater.

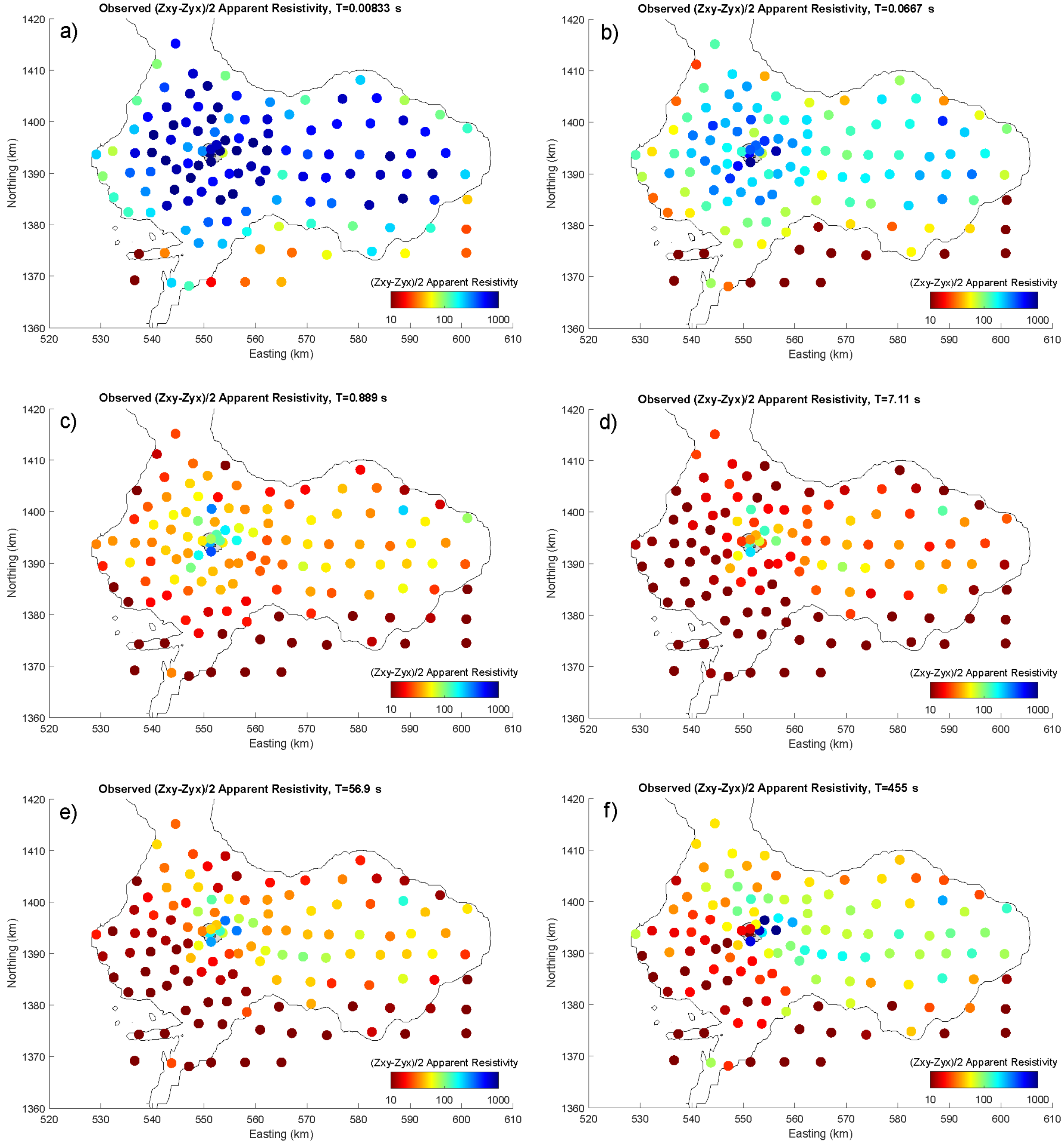

Supplementary Figure S6: Observed invariant tensor impedance (Z_{xy}-Z_{yx}/2) plotted as "spot" diagrams over six widely spaced periods illustrating the apparent resistivity structure for the Mount Erebus/Ross Island MT survey.

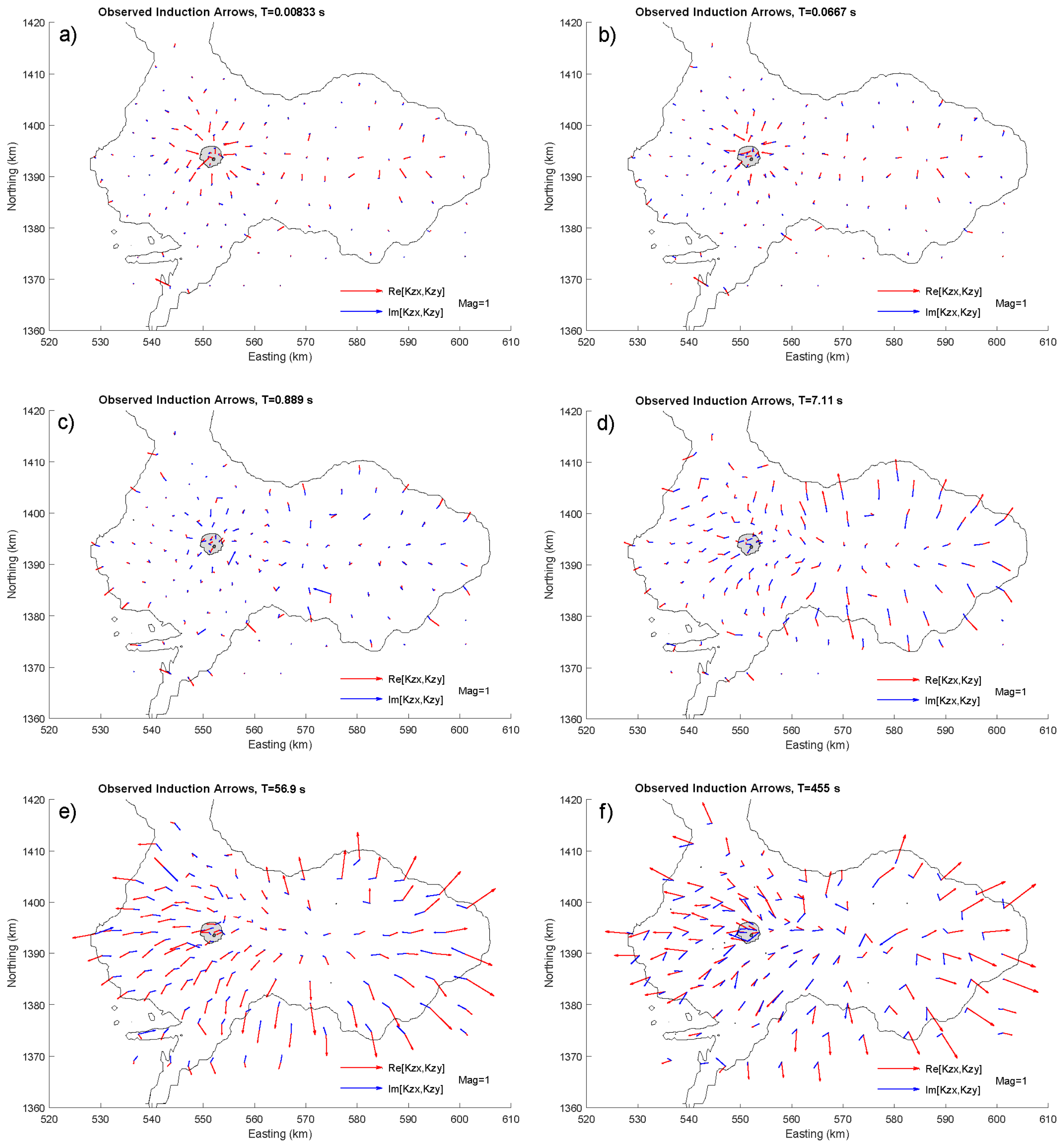

Supplementary Figure S7: Observed vertical magnetic field induction arrows over six widely spaced periods for the Mount Erebus/Ross Island MT survey using the Parkinson convention where real components of arrows point toward conductors (regions of low resistivity).

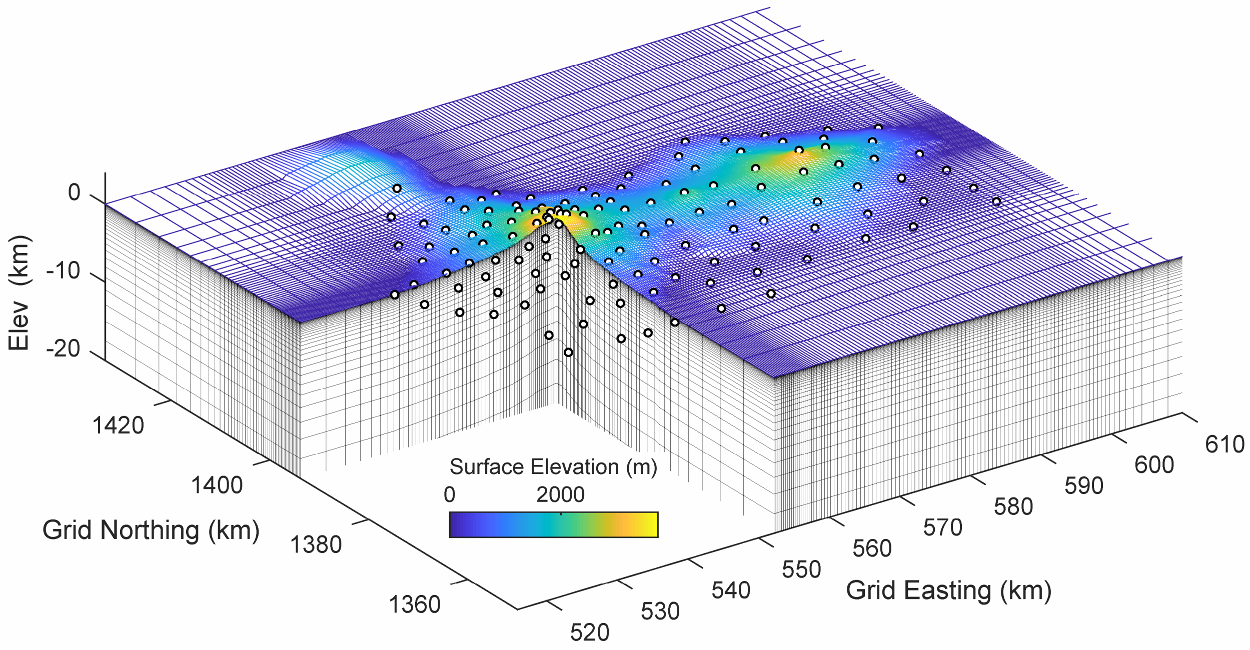

Supplementary Figure S8: Wire frame view of finite element mesh discretization for the 3D inversion[3,4] of the Ross Island/Mount Erebus MT data set. Individual MT stations shown as small circles. Mount Erebus crater summit is at an elevation of 3794 m (12,448 ft).

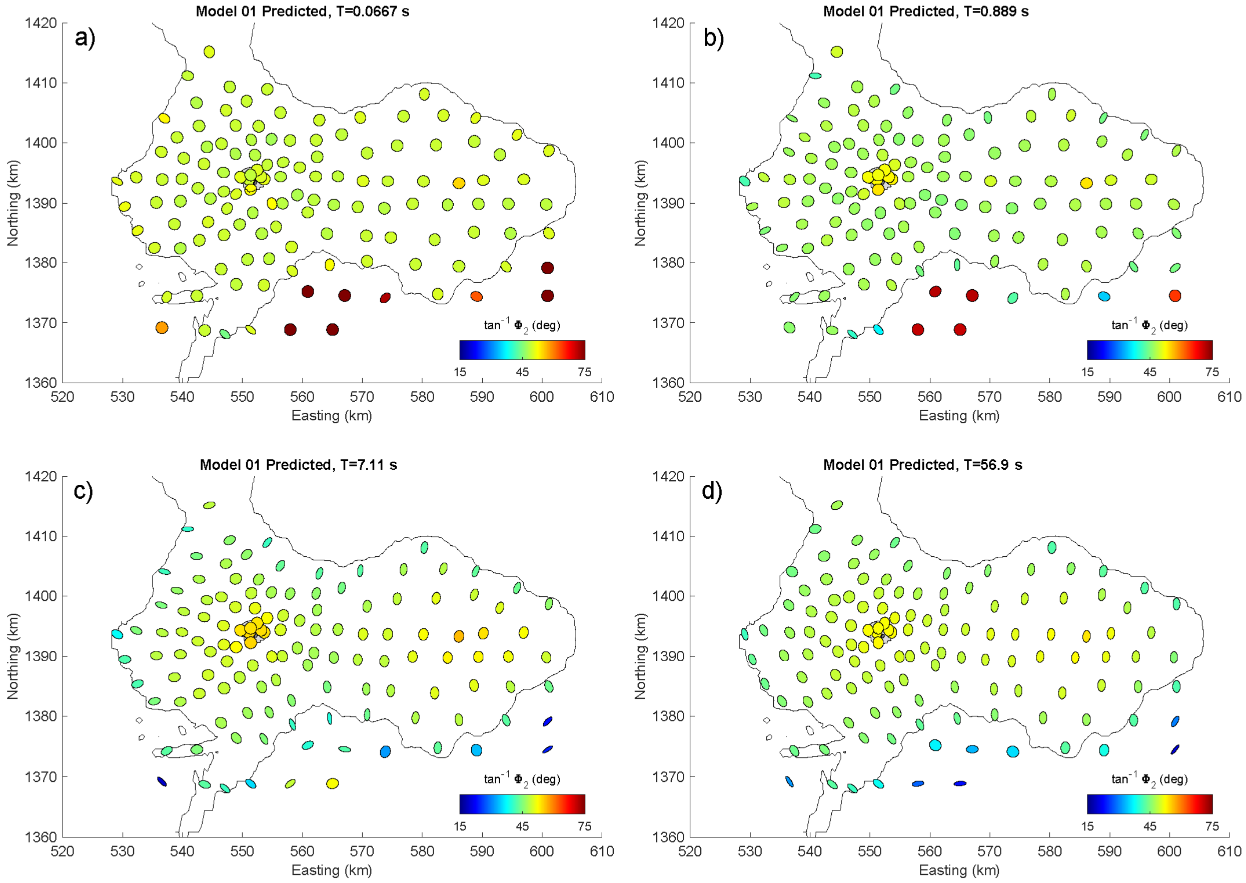

Supplementary Figure S9: Phase tensor plots at four periods computed for the 100 ohm-m finite element starting model including topography and surrounding bathymetry.

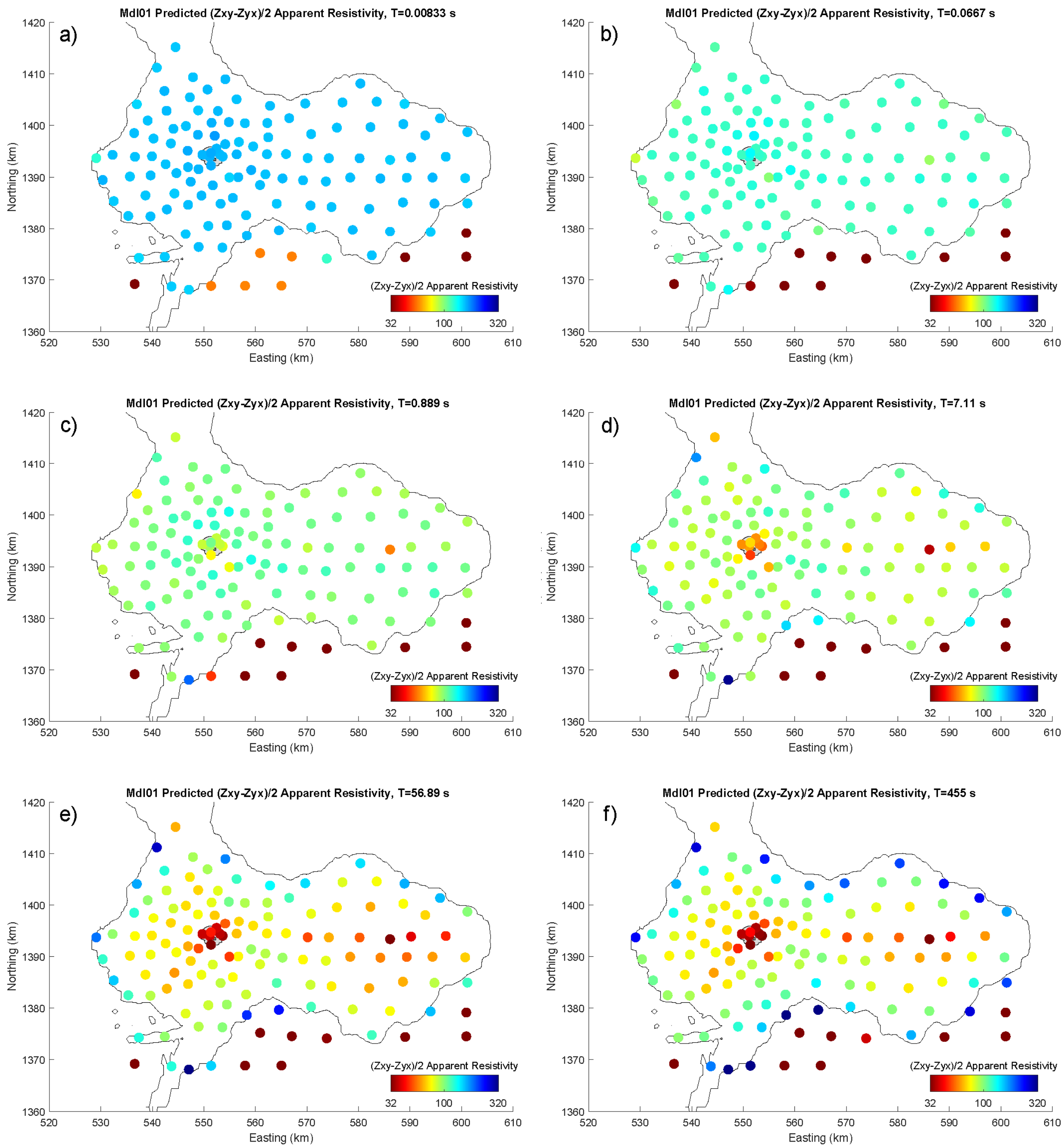

Supplementary Figure S10: Apparent resistivity from invariant tensor impedance [(Z_{xy}-Z_{yx}/2)] plotted as spot diagrams at six periods computed for the 100 ohm-m finite element starting model including topography and surrounding bathymetry. Note the region of low apparent resistivity around the summit crater area. This is due to topography, and not subsurface structure.

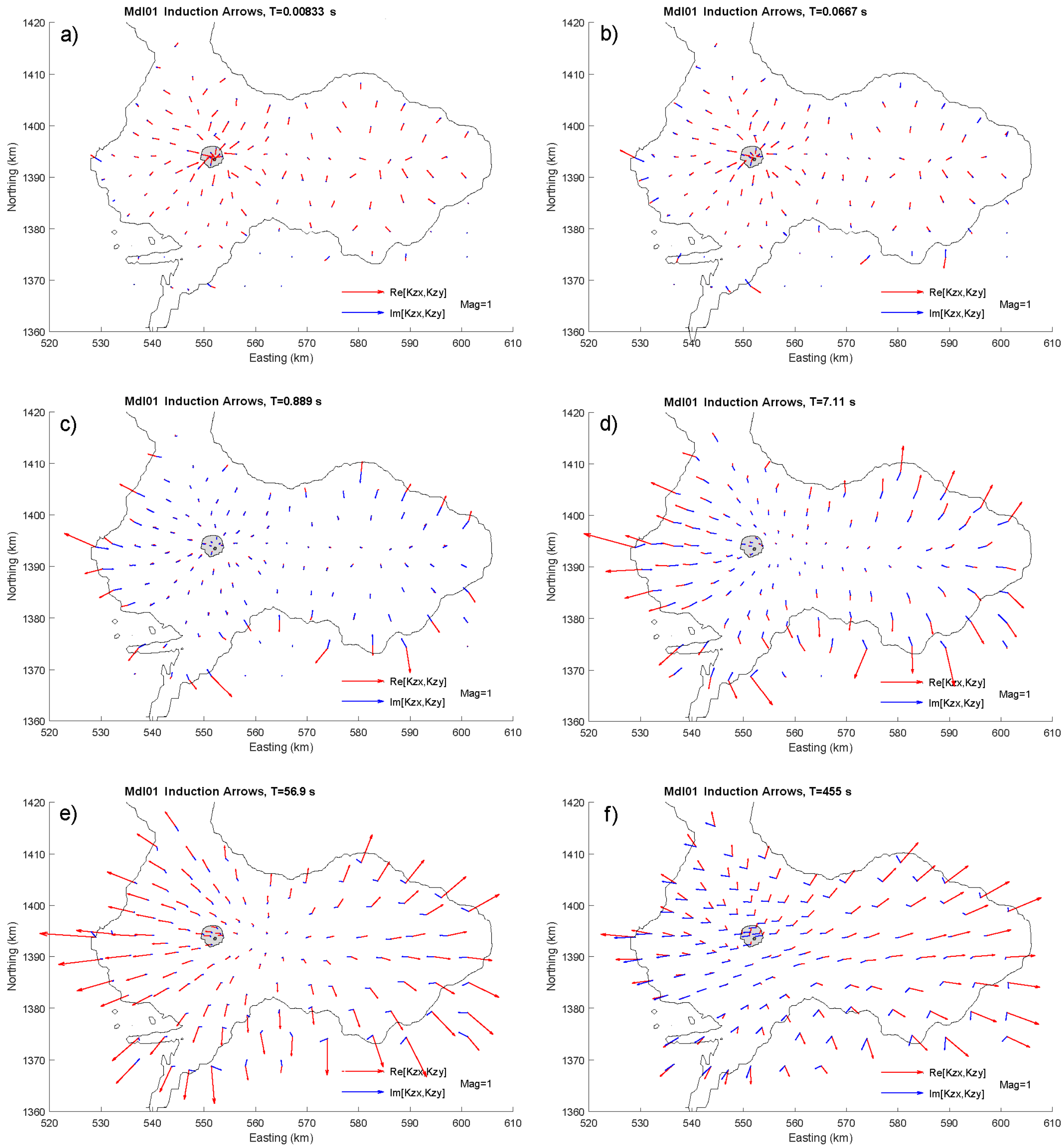

Supplementary Figure S11: Vertical magnetic field induction arrows over six widely spaced periods for the Mount Erebus/Ross Island MT survey computed for the 100 ohm-m finite element starting model including topography and surrounding bathymetry. These variations are due to the topography and surrounding low resistivity seawater (not subsurface structure).

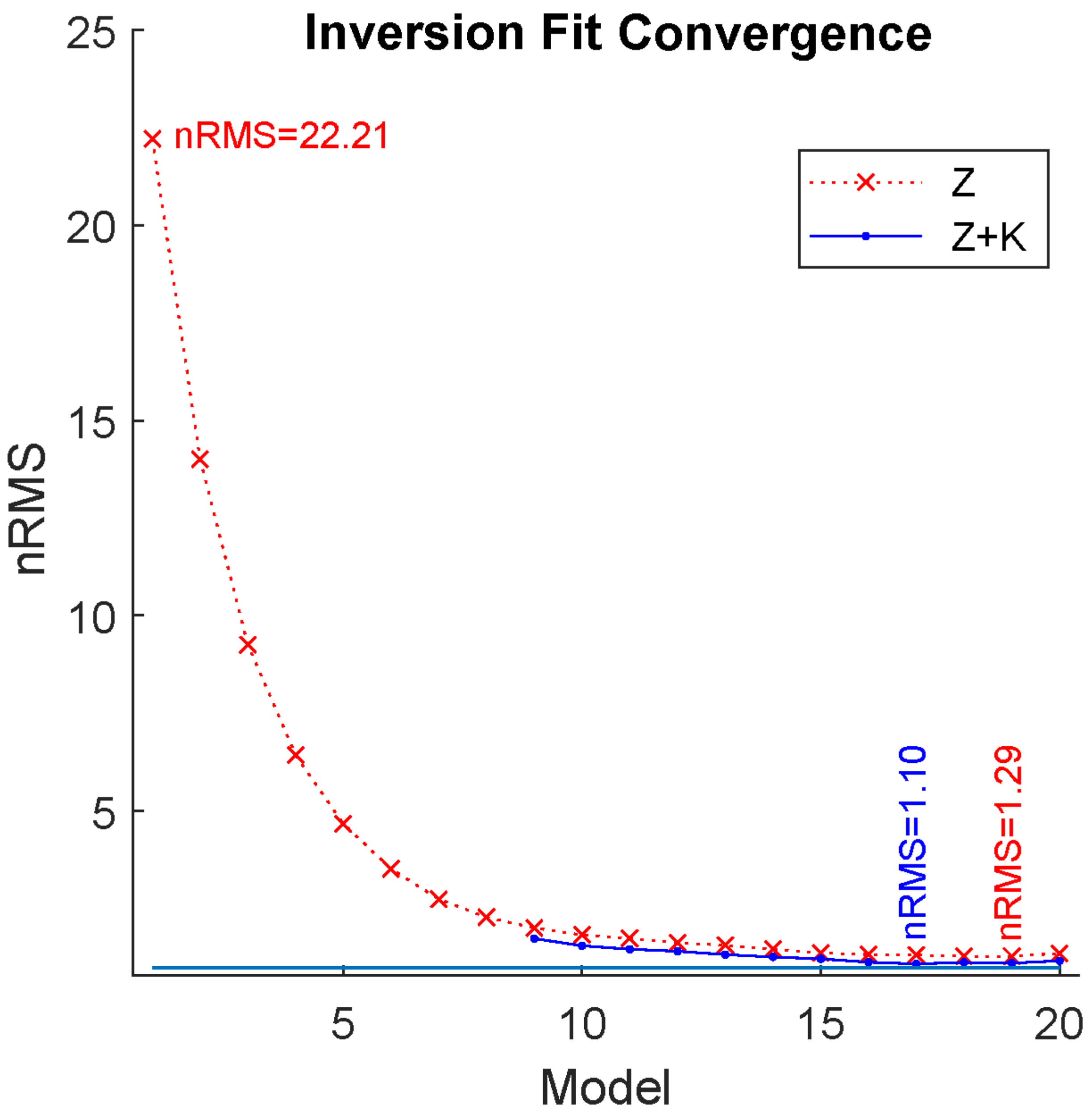

Supplementary Figure S12: Convergence plot of nRMS for finite element inversion from the 100 ohm-m starting model. The red curve results from inversion of tensor impedance Z only, while blue curve shows convergence when limited vertical magnetic field data are added to the inversion. Horizontal medium teal-blue line corresponds to nRMS of unity.

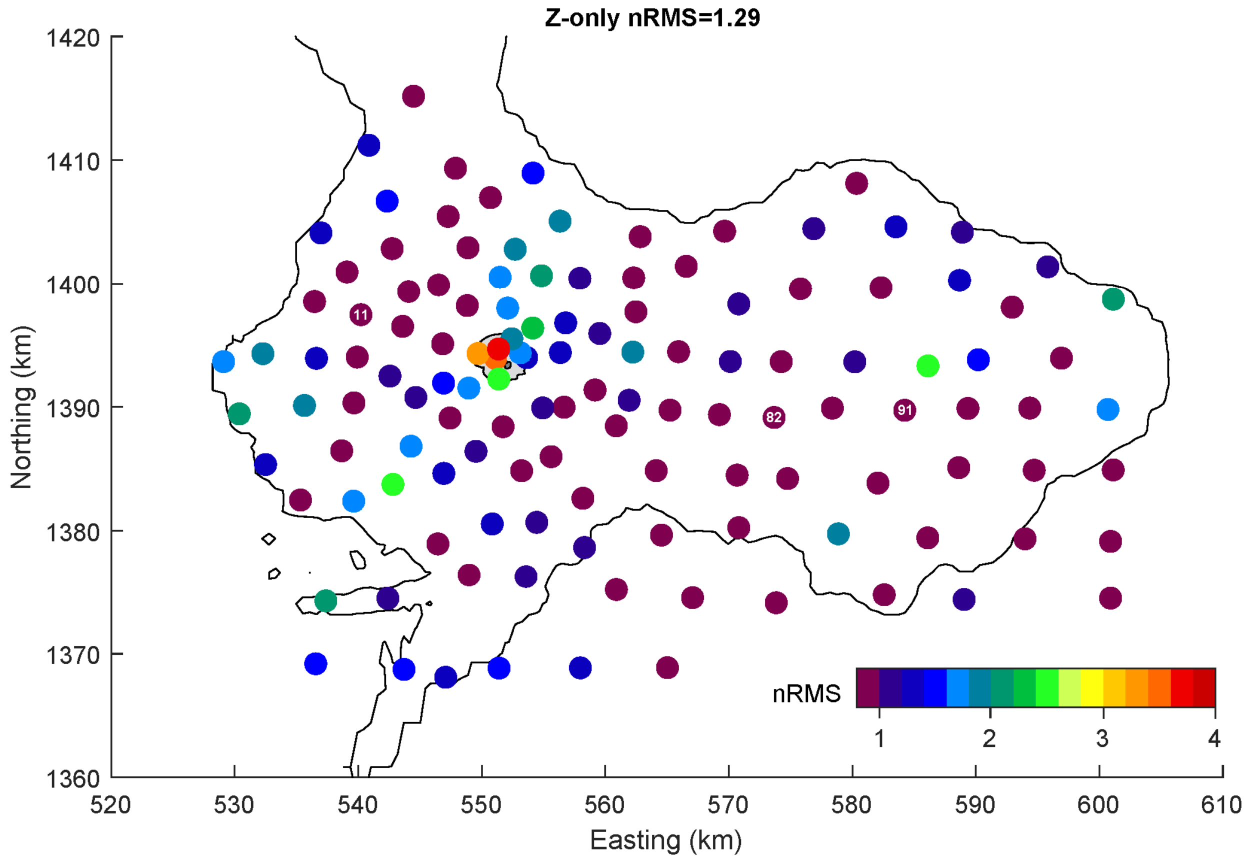

Supplementary Figure S13: nRMS misfit at individual MT stations over Ross Island from our preferred inversion of Figures 2-4. An nRMS of unity is considered ideal, and this value is close to achieved almost everywhere, with the exception of a few sites near the Erebus crater edge where long period data scatter appears larger than the nominal error bars. Site 91 is that of sounding curves in Figure S4 while sites 11 and 82 are those of the sounding curves in Figure S16.

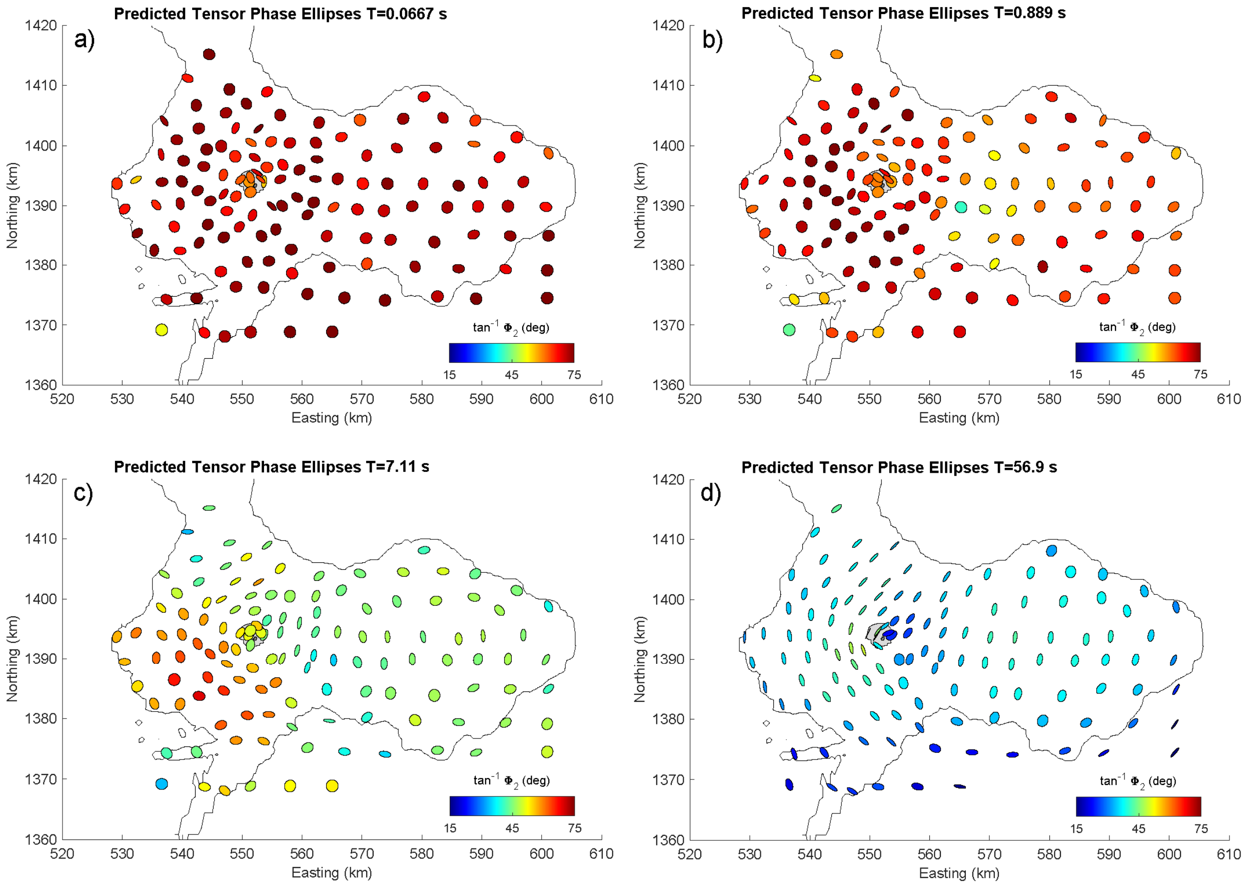

Supplementary Figure S14: Computed impedance phase tensor ellipses for the preferred inversion model of Figures 2-4 at the same four periods as the observed ellipses in Figure S5.

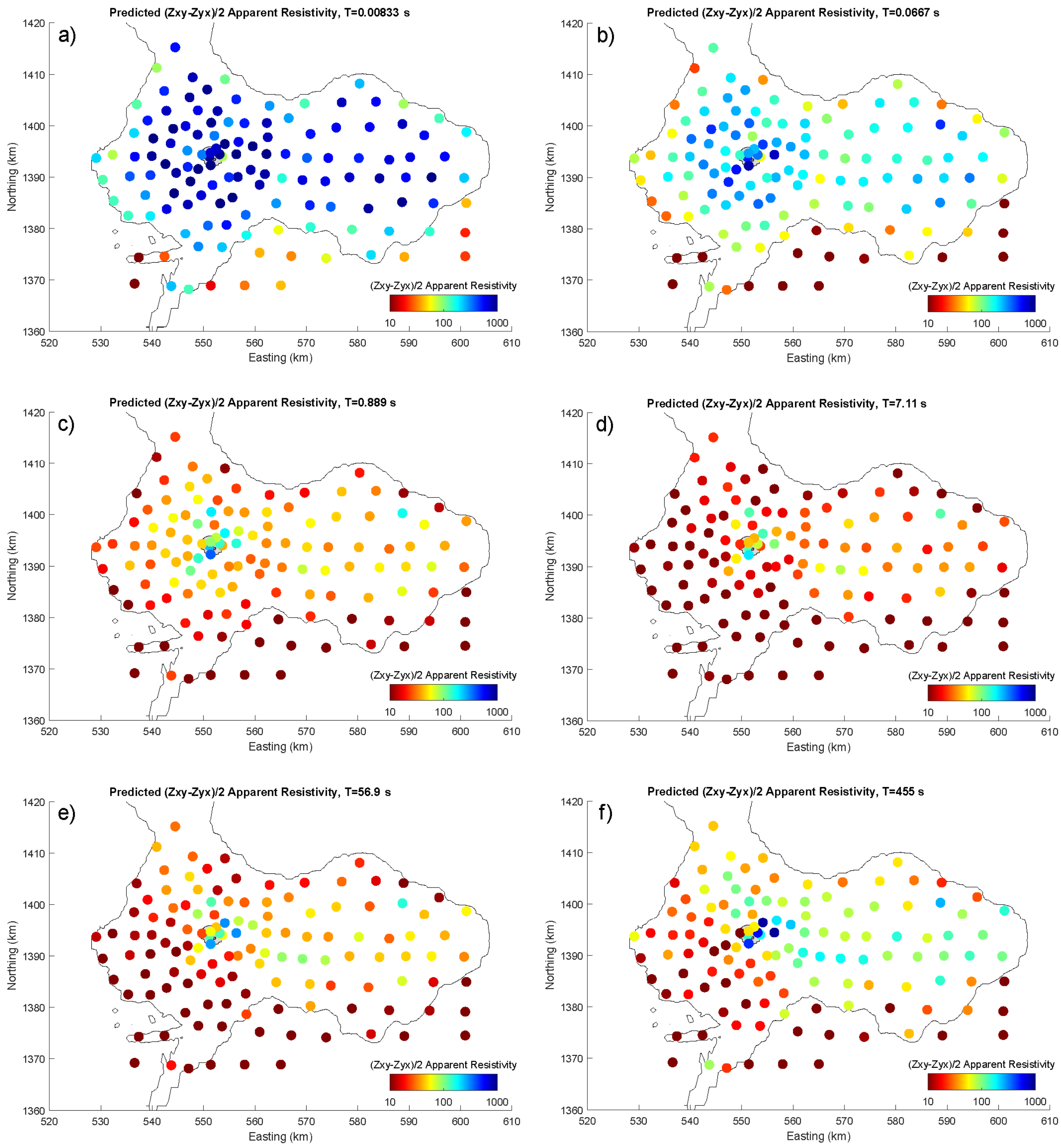

Supplementary Figure S15: Computed apparent resistivity from invariant tensor impedance (Z_{xy}-Z_{yx}/2) plotted as spot diagrams at six periods for the final finite element inversion model of Figures 2-4. Note scale change from Supplementary Figure S10.

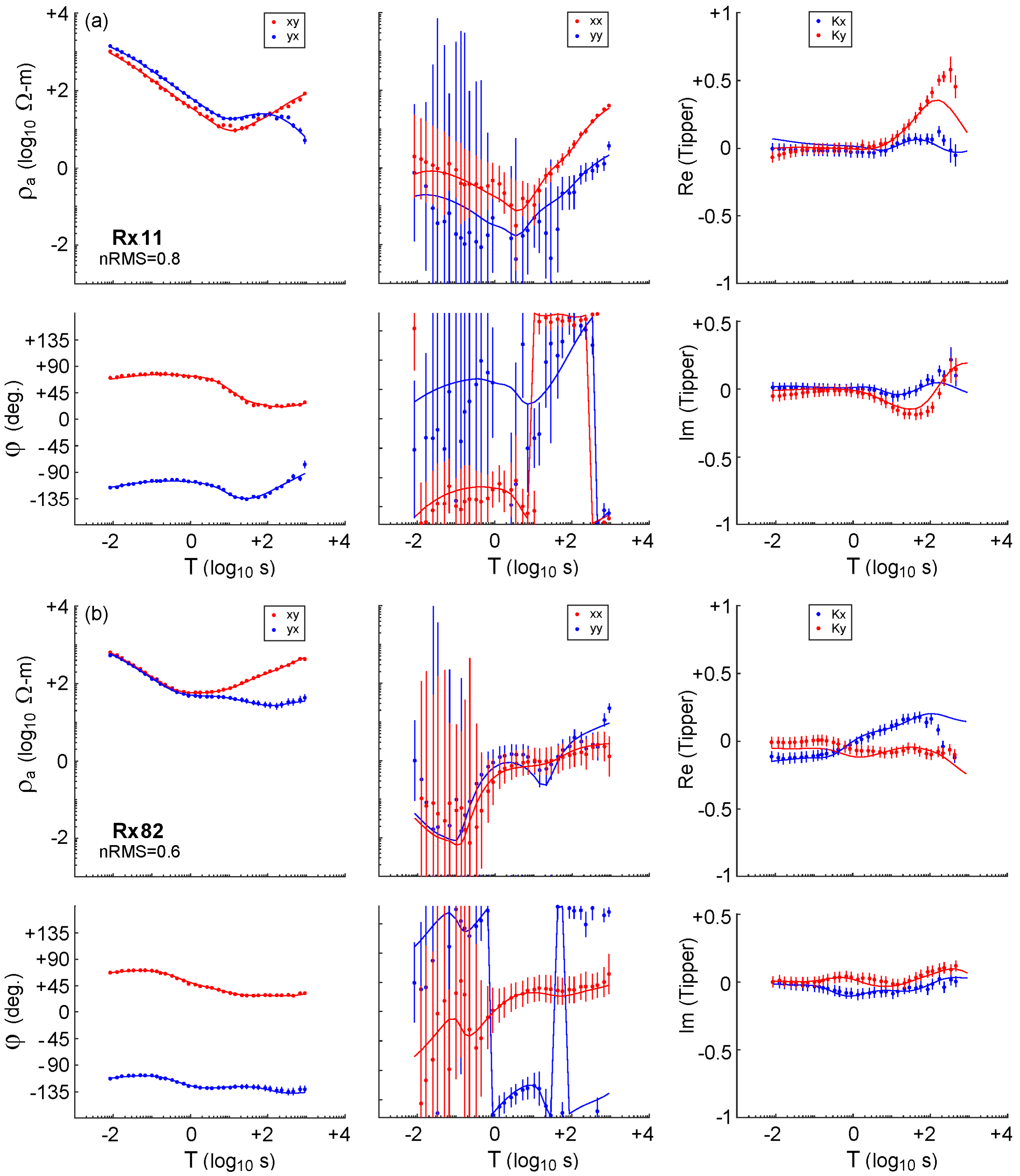

Supplementary Figure S16: Two example apparent resistivity (ρ_a), impedance phase (φ) and vertical magnetic field transfer function soundings from west (a) and east (b) of Erebus summit crater. Sounding Rx numbers are shown on Figure S13. Computed curves are for the impedance-only inversion model presented in the main text. The vertical magnetic transfer function data were not explicitly inverted in this example although agreement is qualitatively good.

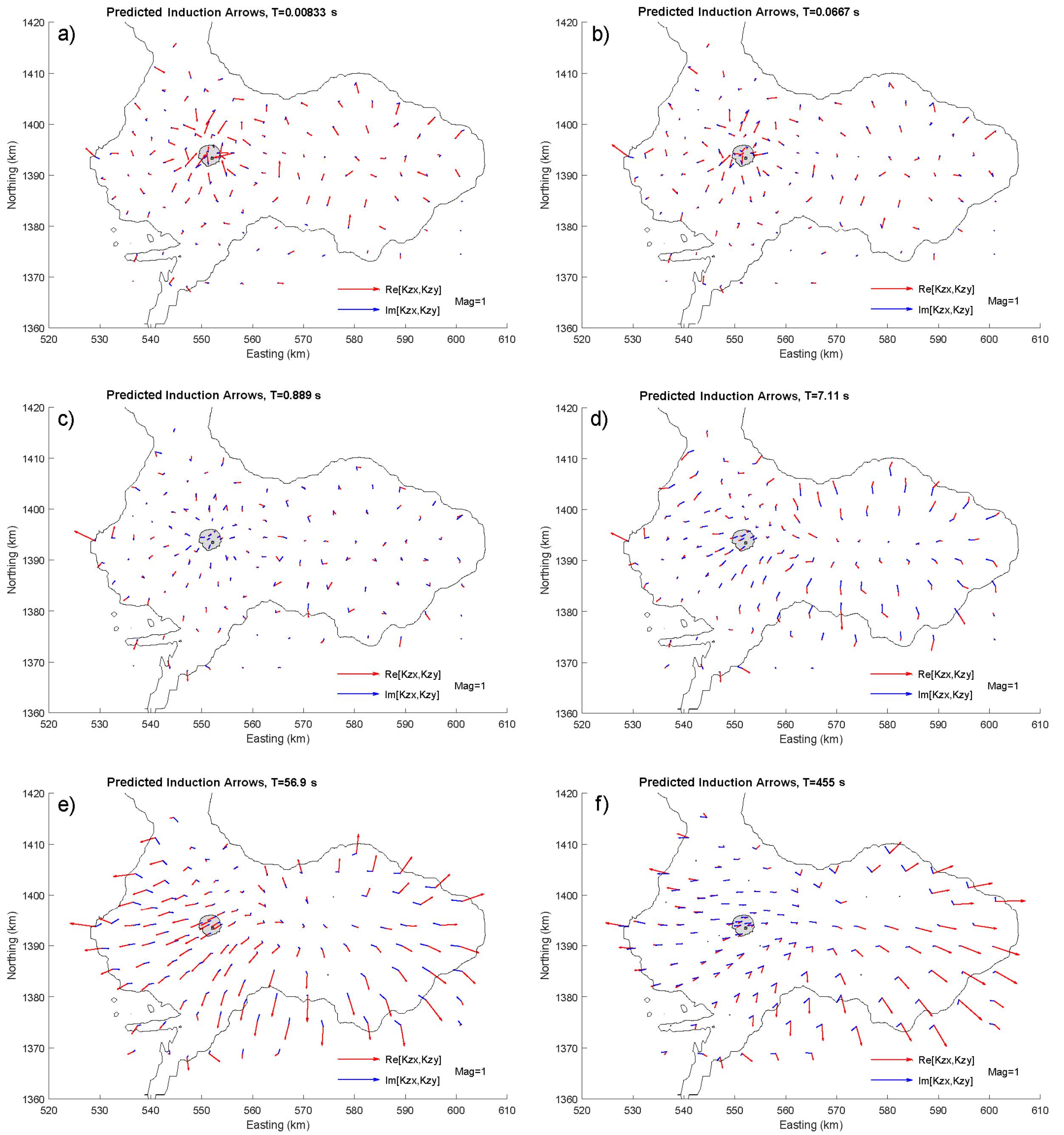

Supplementary Figure S17: Computed vertical magnetic field induction arrows at six periods for the finite element inversion model of Figures 2-4.

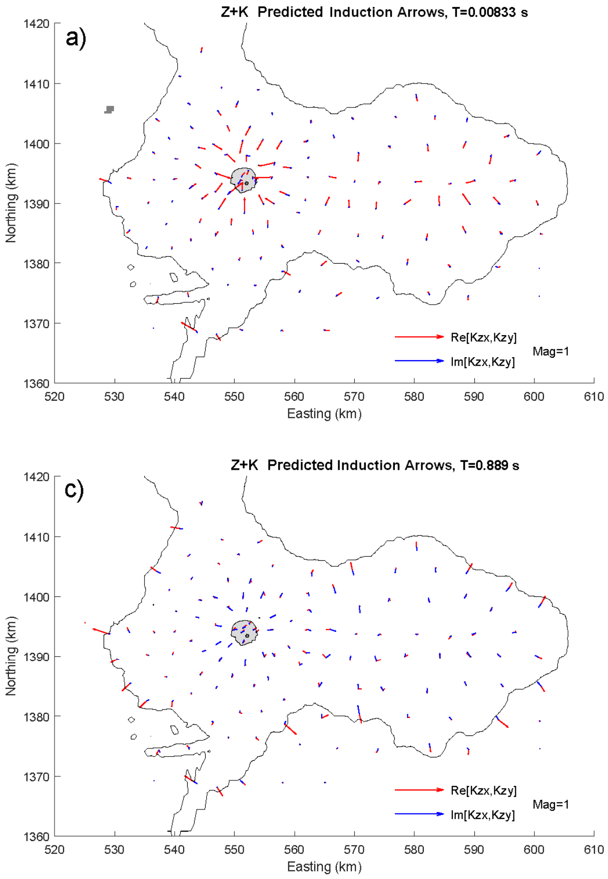

Supplementary Figure S18: Computed vertical magnetic field induction arrows at four periods for the joint Z+K finite element inversion model of Supplementary Figure S19.

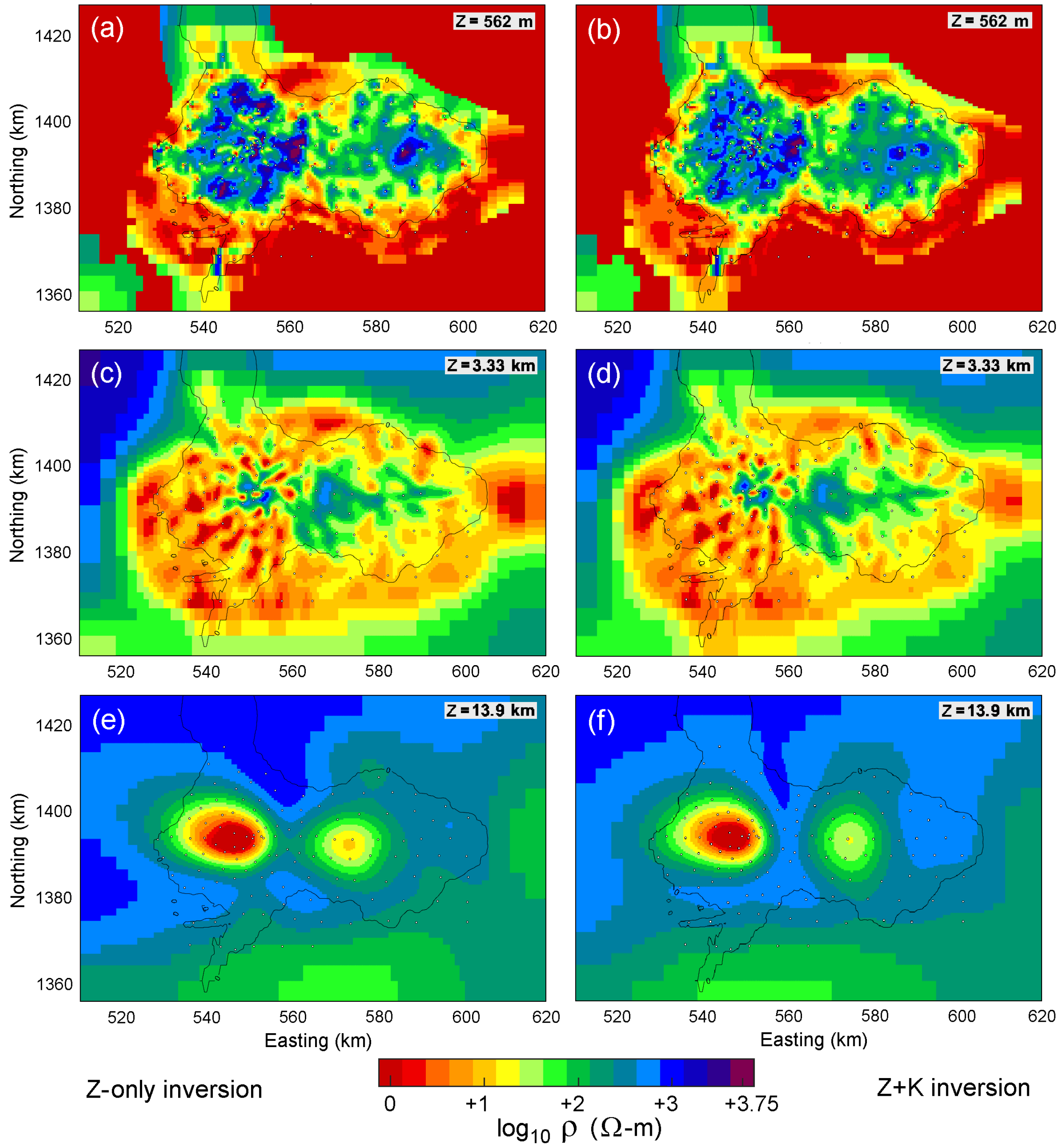

Supplementary Figure S19: Plan views of the shallower portion of the Ross Island MT inversion resistivity (ρ) model comparing Z-only results (left) with Z+K results (right) primarily to assess effect of the vertical magnetic field transfer function on upper to middle crustal structure.

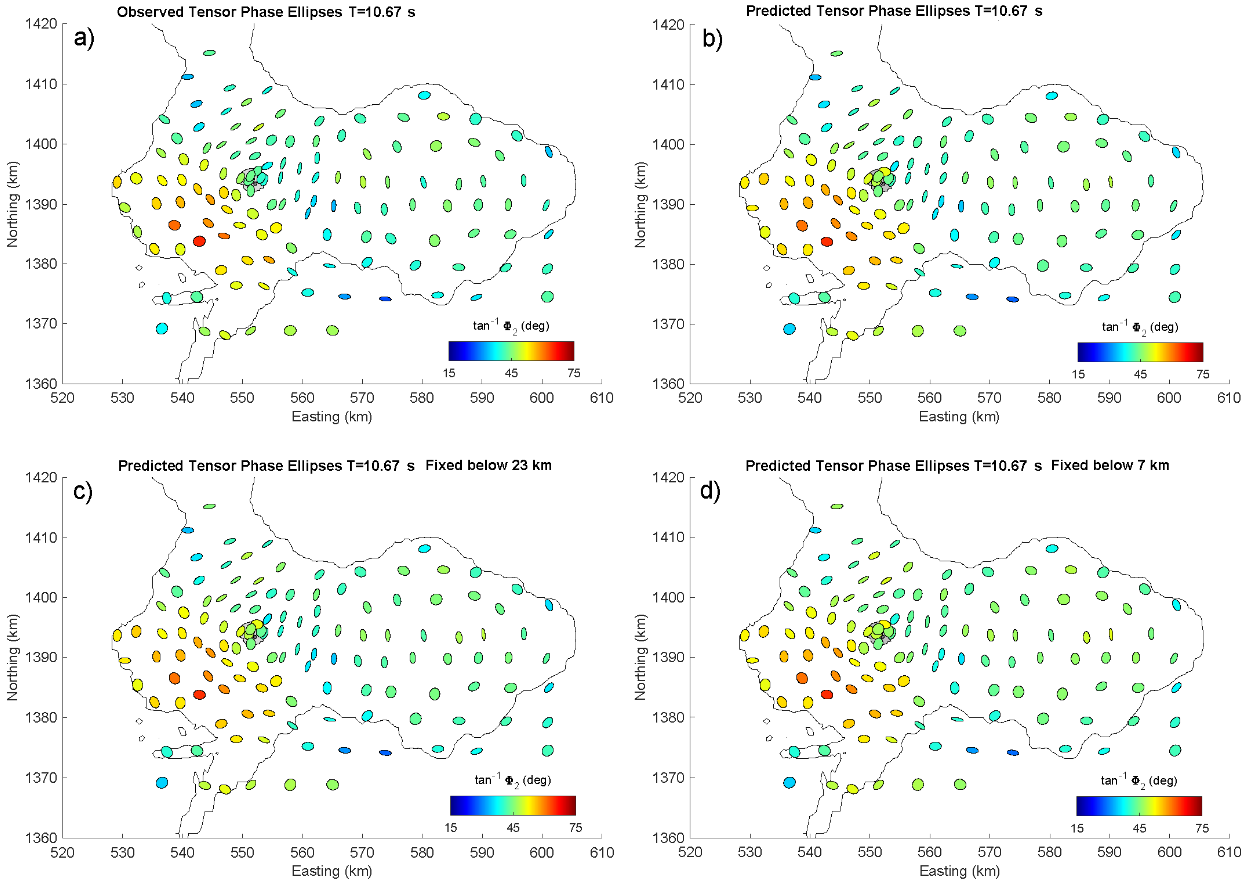

Supplementary Figure S20: Observed phase tensor ellipses at 10.7s (upper left) compared to those computed from the preferred inversion model (upper right) plus from the preferred model truncated below 23 km depth (lower left) and truncated below 7 km depth (lower right).

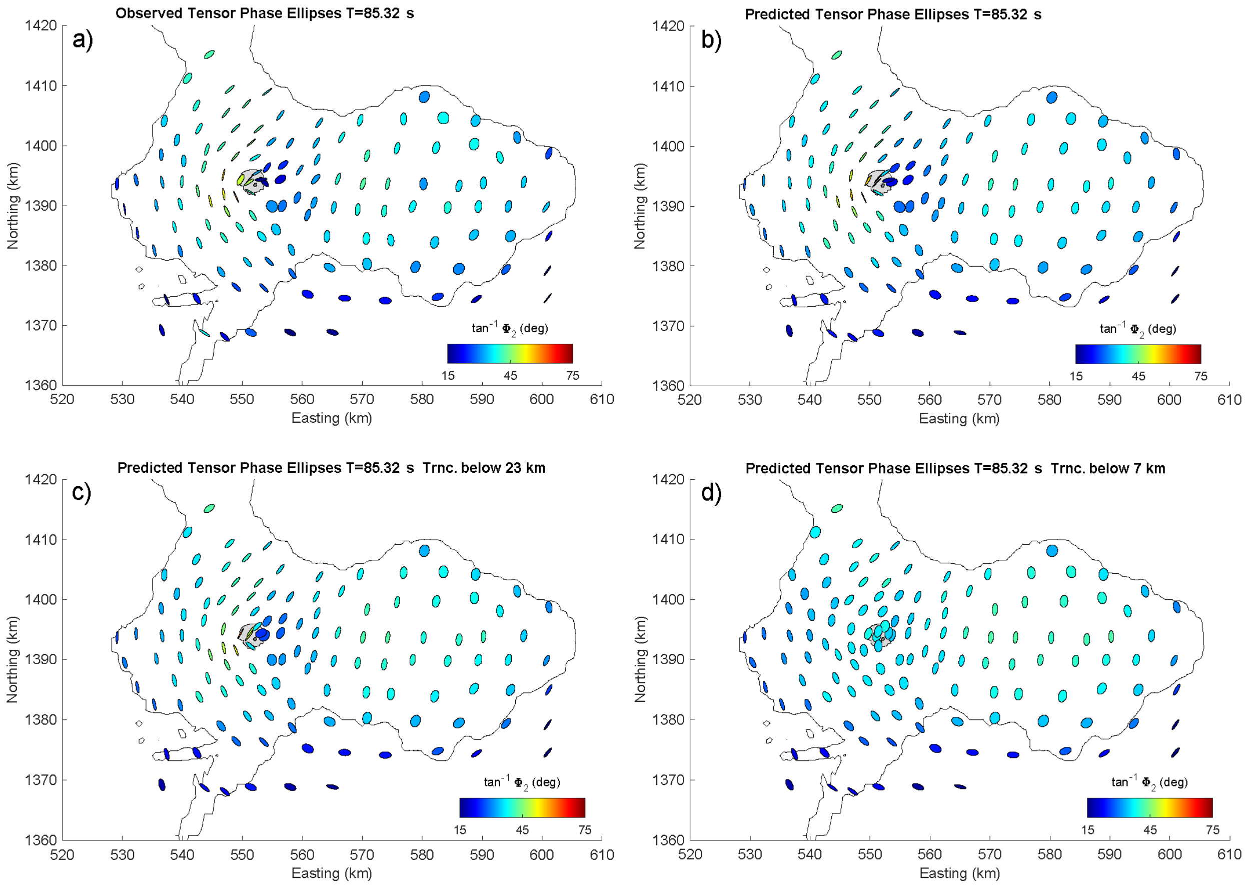

Supplementary Figure S21: Observed phase tensor ellipses at 85s (upper left, a) compared to those computed from the preferred inversion model (upper right, b) plus from the preferred model truncated below 23 km depth (lower left, c) and truncated below 7 km depth (lower right, d).

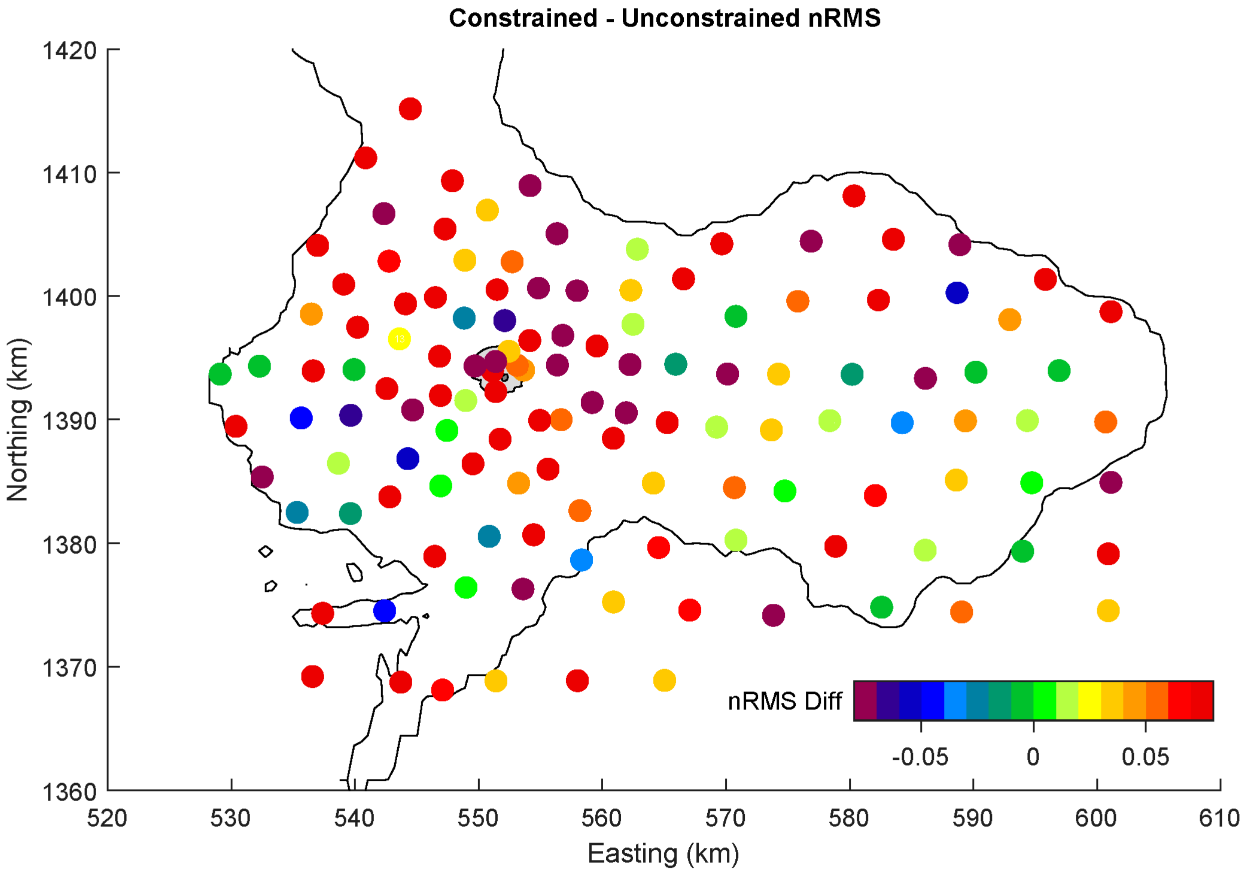

Supplementary Figure S22: Spot diagram displaying differences in nRMS misfit between Moho-constrained and unconstrained inversions. Positive values denote that the unconstrained model fit is closer than the constrained, and vice versa. Total unconstrained nRMS = 1.29 whereas constrained nRMS = 1.33.

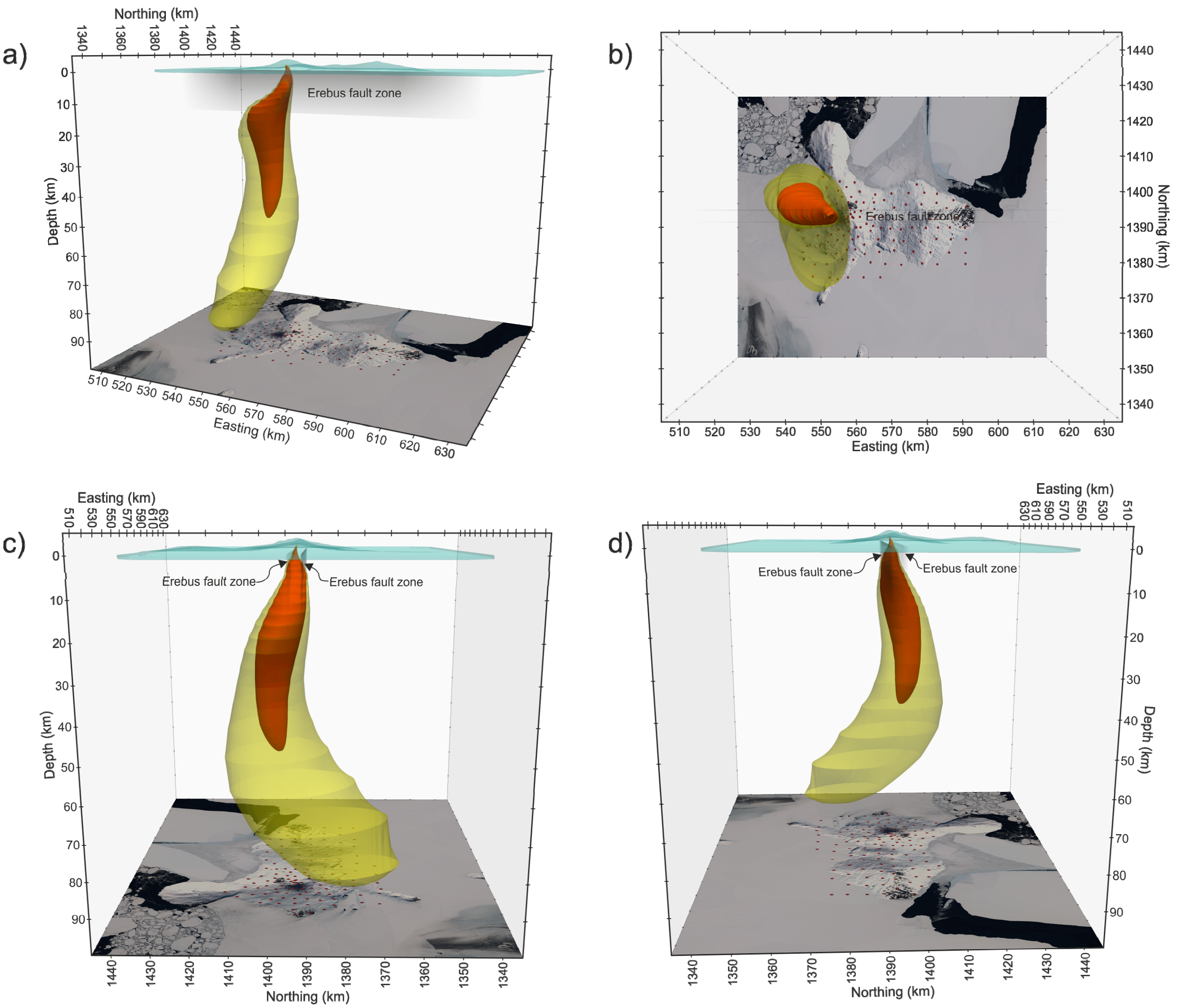

Supplementary Figure S23: Four 3D visualizations of the Mount Erebus MT inversion model: (a), view toward Mercator 330; (b) view from directly over Erebus crater; (c), view toward Mercator 080; (d), view toward Mercator 270. The colour bounds are 5 (red) and 10 (yellow) Ωm. From prior sensitivity test inversion, the depth extent of the low resistivity into the upper mantle is approximately resolved.

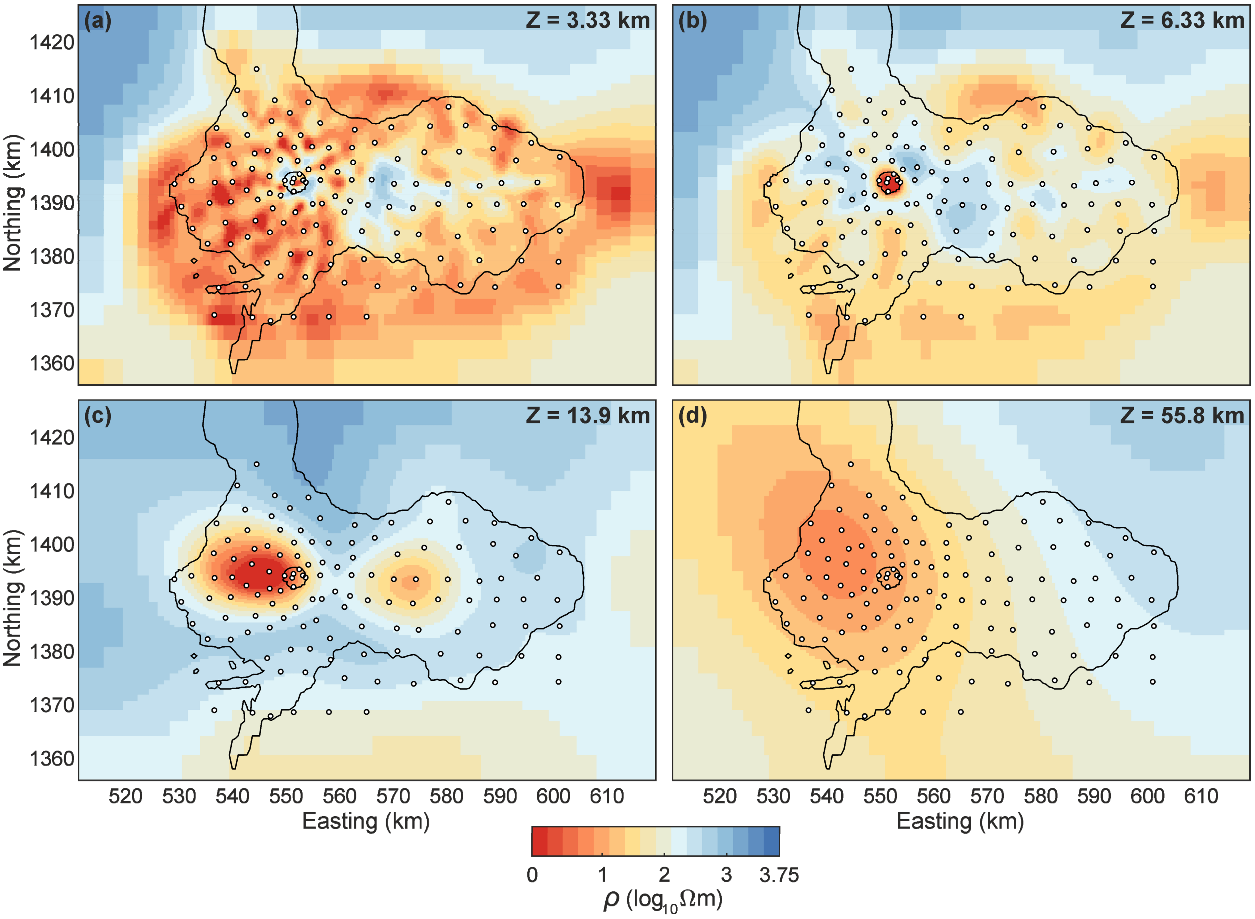

Supplementary Figure S24: Alternate colour map version of Figure 2.

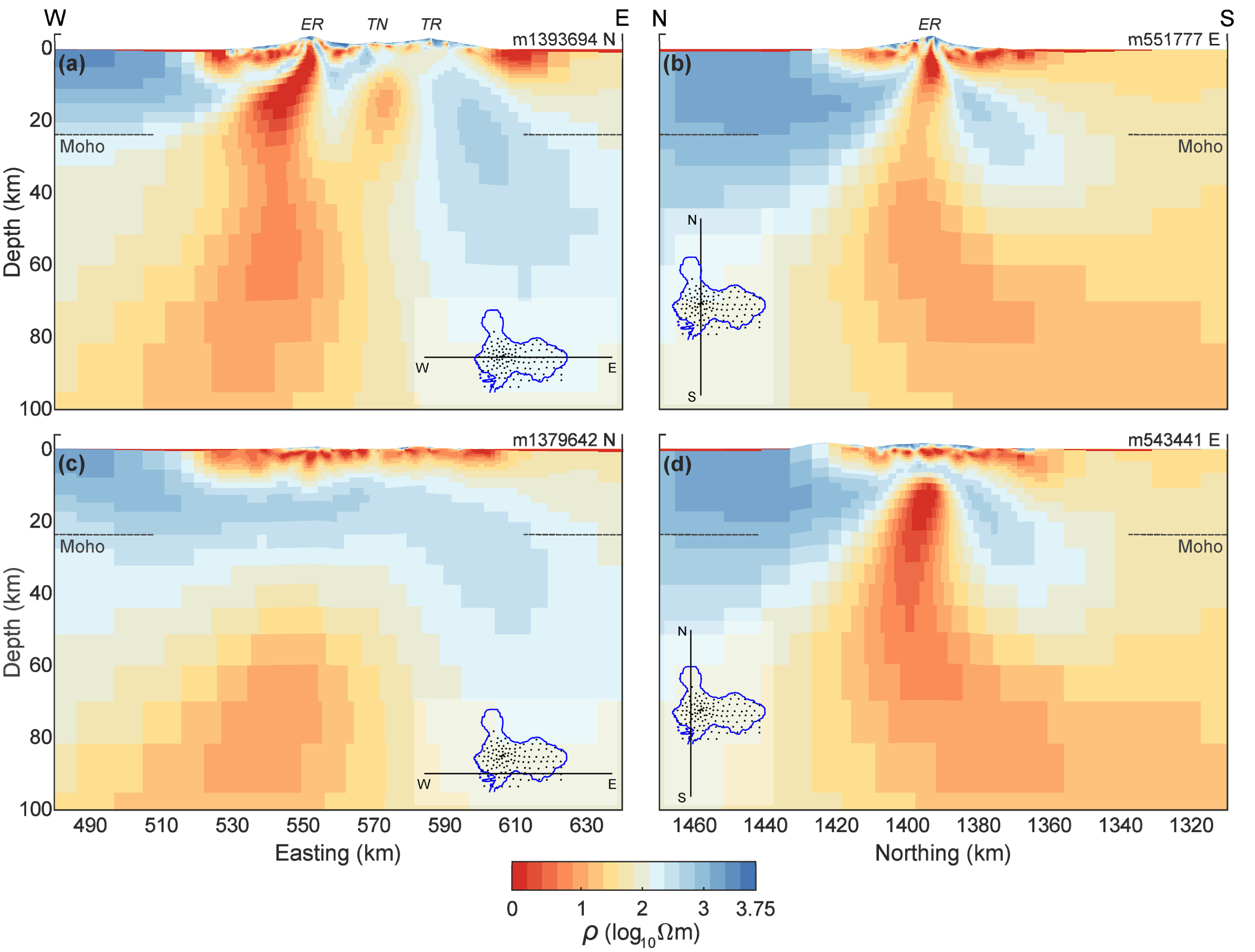

Supplementary Figure S25: Alternate colour map version of Figure 3.

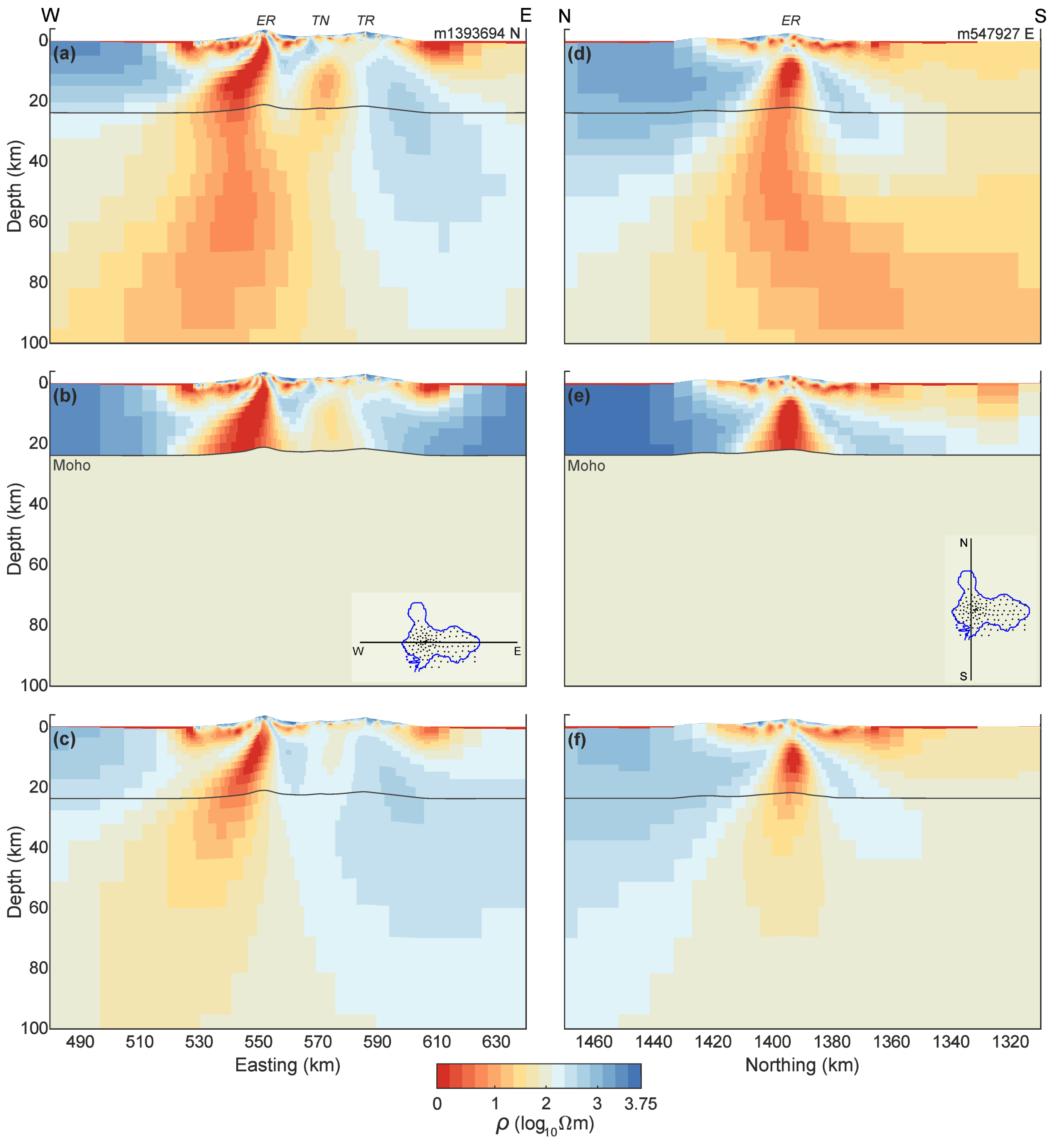

Supplementary Figure S26: Alternate colour map version of Figure 4.
